# Supplementary material for: Mass testing for discovery and control of COVID-19 outbreaks in adult social care: an observational study and cost-effectiveness analysis of 14 805 care homes in England
Source: BMJ Public Health. 2025 Mar 28;3(1):e001376. doi: 10.1136/bmjph-2024-001376 (PMC11962772; doi:10.1136/bmjph-2024-001376)
Supplement: online supplemental table 1 [file bmjph-3-1-s001.docx]

**Supplementary Materials**

**Title: Mass testing for discovery and control of COVID-19 outbreaks in adult social care: an observational study and cost-effectiveness analysis of 14,805 care homes in England**

**Authors:**

Siyu Chen^1†^, Richard Creswell^2†^, Rachel Hounsell^3,6†^, Liberty Cantrell^4^, Sumali Bajaj^5^, Prabin Dahal^3,6^, Lok Hei Tsui^5^, Olumide Kolade^7^, Ma’ayan Amswych^8^, Reshania Naidoo^3,8^, Tom Fowler^7^, Susan Hopkins^7,9^, Kasia Stepniewska^6^, Merryn Voysey^4^, Lisa White^5^, Rima Shretta^3^, Ben Lambert^10,11*^ on behalf of the EY-Oxford Health Analytics Consortium^**^

**Affiliations:**

^1^High Meadows Environmental Institute, Princeton University, USA; Department of Public and Ecosystem Health, College of Veterinary Medicine, Cornell University, USA

^2^Department of Computer Science, University of Oxford, UK

^3^Nuffield Department of Medicine, University of Oxford, UK

^4^Department of Paediatrics, University of Oxford, UK

^5^Department of Biology, University of Oxford, UK

^6^Centre for Tropical Medicine and Global Health, University of Oxford, UK

^7^UK Health Security Agency, London, UK

^8^Ernst & Young LLP, London, UK

^9^NIHR Health Protection Research Unit in Healthcare associated infections and Antimicrobial Use, University of Oxford, UK

^10^Department of Statistics, University of Oxford, UK

^11^Pandemic Sciences Institute, University of Oxford, UK

^†^ These authors contributed equally.

*Corresponding author: [ben.lambert@stats.ox.ac.uk](mailto:ben.c.lambert@gmail.com)

**EY-Oxford Health Analytics Consortium membership list (as listed in a separate file)

Ricardo Aguas, Ma’ayan Amswych, Billie Andersen-Waine, Sumali Bajaj, Kweku Bimpong, Adam Bodley, Liberty Cantrell, Siyu Chen, Richard Creswell, Prabin Dahal, Sophie Dickinson, Sabine Dittrich, Tracy Evans, Angus Ferguson-Lewis, Caroline Franco, Bo Gao, Rachel Hounsell, Muhammad Kasim, Claire Keene, Ben Lambert, Umar Mahmood, Melinda Mills, Ainura Moldokmatova, Sassy Molyneux, Reshania Naidoo, Randolph Ngwafor Anye, Jared Norman, Wirichada Pan-Ngum, Sarah Pinto-Duschinsky, Sunil Pokharel, Anastasiia Polner, Katarzyna Przybylska, Emily Rowe, Sompob Saralamba, Rima Shretta, Sheetal Silal, Kasia Stepniewska, Joseph Tsui, Merryn Voysey, Marta Wanat, Lisa White, Gulsen Yenidogan

**Contents**

[1. Summary of COVID-19 testing policy changes for adult social care in England 4](#_Toc187225589)

[1.1. Early response in adult social care to the COVID-19 pandemic (pre-evaluation period) 4](#_Toc187225590)

[1.2. Symptomatic testing 4](#_Toc187225591)

[1.3. Initiation of asymptomatic testing in care homes 5](#_Toc187225592)

[2. Data resources 6](#_Toc187225593)

[**2.1.** **Testing data** 6](#_Toc187225594)

[**2.2.** **Death data** 6](#_Toc187225595)

[**2.3.** **Care home characteristics** 6](#_Toc187225596)

[**2.4.** **COVID-19 community prevalence** 6](#_Toc187225597)

[**2.5.** **SARS-CoV-2 lineages** 7](#_Toc187225598)

[**2.6.** **Vaccination** 7](#_Toc187225599)

[**2.7.** **Regional population** **structure** 7](#_Toc187225600)

[3. Data cleaning 8](#_Toc187225601)

[3.1. Interpolation of time-varying care home data 8](#_Toc187225602)

[3.2. Vaccination level 8](#_Toc187225603)

[3.3. Merging of testing data and deaths data 8](#_Toc187225604)

[3.4. Merging of testing/deaths data with other covariates 8](#_Toc187225605)

[4. The association between care home characteristics and reported testing intensity of LFD and PCR tests among residents and staff 8](#_Toc187225606)

[5. The association between outbreak size when they were initially uncovered and care home characteristics in outbreak discovery 9](#_Toc187225607)

[6. The association between subsequent outbreak sizes during outbreaks and care home characteristics in outbreak control 11](#_Toc187225608)

[7. The association between COVID-19-related mortality and care home characteristics 12](#_Toc187225609)

[8. Deaths averted under hypothetical testing scenarios 12](#_Toc187225610)

[9. Deaths averted across testing scenarios 12](#_Toc187225611)

[10. Deaths averted after increasing staff numbers scenarios 12](#_Toc187225612)

[11. Test volumes, costs, and cost effectiveness 12](#_Toc187225613)

[11.1. Data processing 12](#_Toc187225614)

[11.2. Cost-effectiveness analysis 13](#_Toc187225615)

[12. Study limitations 13](#_Toc187225616)

[Figure S1. Example resident reported test intensity with LFDs and PCR in small care homes. 15](#_Toc187225617)

[Figure S2. Example resident reported test intensity with LFDs and PCR in mid-sized care homes. 15](#_Toc187225618)

[Figure S3. Associations between the number of positives and the number of COVID-19-related total deaths, by primary client type served by care homes. 16](#_Toc187225619)

[Figure S4. Projected COVID-19-related total deaths under counterfactual testing scenarios: sensitivity analysis. 16](#_Toc187225620)

[Figure S5. Projected COVID-19-related total deaths under counterfactual staff scenarios: sensitivity analysis. 16](#_Toc187225621)

[Table S1. Description of testing policy in care homes in England. 17](#_Toc187225622)

[Table S2. Number of LFDs distributed and PCR tests registered in England as part of the adult social care testing service (during the evaluation period). 17](#_Toc187225623)

[Table S3. Total financial cost of the adult social care testing service for England (during the evaluation period). 17](#_Toc187225624)

[Table S4. Regression results for reported testing intensities (PCR and LFD tests) among residents and staff. All sets of regression results represent estimates for linear models fitted using ordinary least squares. The figures in parentheses represent 95% confidence intervals for the coefficient estimates. 17](#_Toc187225625)

[Table S5. Naïve regressions of numbers of positives in residents on lagged resident and staff tests. 19](#_Toc187225626)

[Table S6. Outbreak discovery in residents – sensitivity analyses. 19](#_Toc187225627)

[Table S7. Outbreak discovery in staff – sensitivity analyses. 21](#_Toc187225628)

[Table S8. Analysis of deviance for the resident outbreak discovery model. 24](#_Toc187225629)

[Table S9. Analysis of deviance for the staff outbreak discovery model. 24](#_Toc187225630)

[Table S10. Outbreak discovery in residents – date sensitivity analyses. 25](#_Toc187225631)

[Table S11. Outbreak discovery in staff – date sensitivity analyses. 27](#_Toc187225632)

[Table S12. Regression results for determinants of weekly outbreak size in residents and staff (measured by number of positives) during outbreaks: sensitivity analysis. 29](#_Toc187225633)

[Table S13. Regression results for determinants of weekly outbreak size in residents (measured by number of positives) during outbreaks: time-period sensitivity analysis. 31](#_Toc187225634)

[Table S14. Regression results for determinants of weekly outbreak size in staff (measured by number of positives) during outbreaks: time-period sensitivity analysis. 32](#_Toc187225635)

[Table S15. Summary of the costs and cost effectiveness of the testing programme in care homes compared with hypothetical changes in the testing volume for the financial years FY21 and FY22. 34](#_Toc187225636)

[Table S16. Data inputs and assumptions for the testing service in adult social care 35](#_Toc187225637)

[Supplementary Materials – References 35](#_Toc187225638)

**1.** **Summary of COVID-19 testing policy changes for adult social care in England**

## Early response in adult social care to the COVID-19 pandemic (pre-evaluation period)

- On 25 February 2020, Public Health England (PHE) issued initial COVID-19 guidance for social care settings and advised that “based on the data available at the time...it is very unlikely that anyone receiving care in a care home or the community will become infected”, and “there is no need to do anything differently in any care setting at present” and also “if staff, member of the public or resident becomes unwell in the workplace and has travelled to China or other affected countries, the unwell person should be removed to an area which is at least 2 metres away from other people. If possible, find a room or area where they can be isolated behind a shut door, such as a staff office. If it is possible to open a window, do so for ventilation..”^1^
- On 19 March 2020, NHS England published a letter detailing the “COVID-19 Hospital Discharge Service Requirements”, to request immediate action on discharging patients who were medically fit to leave hospital, to free up capacity for COVID-19 cases.^2^ There was no national policy requirement to test patients before being discharged outlined.^3^
- On 2 April 2020, the Department of Health and Social Care (DHSC), the Care Quality Commission (CQC), and NHS England jointly published new guidance for care homes, including that a negative test result for COVID-19 was not required prior to admission into care homes and that testing was offered for up to five initial possible cases to confirm outbreaks where there was more than one symptomatic resident. It advised against visitors except in exceptional situations and “care as normal” for individuals without symptoms.^4^. About the isolation policy, reference 4 stated that the self-isolation period for residents is now 10 days. It is possible for residents to end their self-isolation earlier than 10 days, subject to a risk assessment and negative rapid lateral flow test results. All residents should self-isolate for 10 days if:
  - they test positive for COVID-19, regardless of whether they are symptomatic or asymptomatic
  - they have symptoms of COVID-19 and are awaiting a PCR test result to confirm their COVID-19 status (if they receive a negative PCR test, they no longer need to self-isolate - however, they may have another illness, and clinical advice should be sought)
  - they are a contact of a person with COVID-19 and are required to self-isolate regardless of vaccination status - see section 1.8 for more information
  - they have been discharged following an unplanned overnight hospital stay
  - following a risk assessment, they are required to self-isolate due to one of the reasons outlined in section of reference 4.

## Symptomatic testing

- On 15 April 2020, DHSC announced a new plan to test (with polymerase chain reaction, PCR) every social care worker who needed one; it also included a plan to test, as a matter of course, all symptomatic care home residents and all patients discharged from hospital prior to being sent to a care home.^5 6^ On 16 April 2020, the NHS published new guidance stating that all patients discharged from a hospital to a care home must be tested for COVID-19, regardless of whether they were residents of the care home previously.^7^ Reference 5 stated “some care providers will be able to accommodate these individuals through effective isolation strategies or cohorting policies. If appropriate isolation/cohorted care is not available with a local care provider, the individual’s local authority will be asked to secure alternative appropriate accommodation and care for the remainder of the required isolation period. The government has made £1.3 billion available to support enhanced discharge from the NHS, and this funding can be drawn on for this alternative provision. We expect local authorities to work together with the NHS to put this approach into practice, in accordance with the NHS Discharge Requirements.”
- On 23 April 2020, DHSC announced that PCR testing would be made available to all essential workers in England and members of their households who were showing symptoms of COVID-19.^8^ However, personal care assistants and unpaid carers were not added to the list of essential workers until the beginning of May 2020.^9^ Reference 8 stated that “booking the test has been made simpler via a new online system. From today, employers can [register and refer self-isolating staff](https://www.gov.uk/guidance/coronavirus-covid-19-getting-tested#arrange-a-test), and from tomorrow employees will be able to [book a test directly for themselves or members of their household](https://www.gov.uk/guidance/coronavirus-covid-19-getting-tested#arrange-a-test) who are experiencing symptoms – a high temperature or new continuous cough.”
- On 28 April 2020, DHSC announced that any individuals in England with COVID-19 symptoms who had to leave home to go to work or were aged 65 years or more were able to get tested via PCR [10]. Alongside this, it was announced that testing was being rolled out for all asymptomatic NHS and social care staff and care home residents.^10^ Reference 10 stated that “This will mean people who cannot work from home and those aged 65 and over can know for sure whether they have coronavirus and need to continue isolating.”
- On 11 May 2020, DHSC launched a new portal via which care homes could arrange PCR testing for COVID-19; while all symptomatic and asymptomatic care home staff and residents in England were eligible for testing, it was prioritised for care homes that looked after the over-65s or those with dementia.^11 12^ This was reiterated in the government’s COVID-19 recovery strategy, published on the same day, which stated that all care homes for those aged 65 years or more would be offered PCR testing by 6 June 2020.^13^
- On 18 May 2020, DHSC announced that anyone in the UK who had COVID-19 symptoms was now eligible for a COVID-19 PCR test, with the government aiming to expand total testing capacity to 200,000 tests per day.^14^
- On 21 May 2020, DHSC announced the offer of antibody tests to patients and health and social care staff in England. These tests were made available in a phased manner, but little detail was provided about how the testing service would work in social care, stating that the government would “agree with local leaders the best place in the country to start” and would “work with them to decide how this is implemented”.^15^

## Initiation of asymptomatic testing in care homes

- On 7 June 2020, DHSC announced that whole care-home testing (using PCR tests, as lateral flow device (LFD) tests were yet to be commonly available) for all residents and asymptomatic staff was to be expanded to cover all remaining adult care homes, including care homes catering for adults with learning disabilities or mental health issues, physical disabilities, acquired brain injuries, and other categories for adults aged less than 65 years.^16^ The government also announced that COVID-19 tests had been offered to every care home for the elderly or those with dementia.^17^ At that time, since the launch of whole care-home testing, the government stated it had provided 1,071,103 test kits to 8984 care homes and that they were able dispatch more than 50,000 test kits per day.^17^ In a further announcement on 8 June 2020, it was stated that a new social care sector COVID-19 support taskforce was to be established, to continue supporting the care sector and prevent further transmission.^18^
- On 3 July 2020, staff and residents in care homes for the over-65s and those with dementia were to receive regular, repeat PCR testing for COVID-19 from the following week, as part of a new social care testing strategy.^19^
- From 13 July 2020, enhanced outbreak testing (involving PCR and LFD tests) for care homes was due to be rolled out by the end of the month to all homes that had registered.^20^ However, the regular retesting of whole care homes was unable to proceed as quickly as had been anticipated, due to a variety of factors, including increased demand for testing and unexpected delays; revised timelines were therefore published.^21^ This resulted in individuals in care homes for those aged ≥65 years or for people with dementia having the first of their regular PCR retests by 7 September 2020, with the remaining care homes (for people younger than 65 years) able to register for PCR retesting from 31 August 2020.^21^
- From 23 December 2020, additional twice-weekly rapid LFD testing was introduced for care home staff (or daily testing in the event of identifying a positive case) supported by funding announced by DHSC on 23 December 2020.^22^
- From February 2021 to December 2021, individuals were no longer required to undertake a PCR test for a period of 90 days, had they received a prior positive PCR test.^23^
- In August 2021, care home staff who were double-vaccinated but noted to be a close contact of a positive case were able to continue attending work (with relevant risk assessment in place), having obtained a confirmatory negative PCR test and undertaking daily LFD tests for 10 days that were all negative.^24^
- In December 2021, staff testing with LFDs increased further, to three times a week, in response to the emergence of the Omicron variant.^25 26^
- In January 2022, confirmatory PCR tests following a positive LFD result were temporarily suspended due to the confidence that a positive LFD result was indicative of an individual having COVID-19.^27^
- A further change to the testing regime came into effect on 16 February 2022, when staff testing moved to a pre-shift LFD for all staff on days that they were working, with the removal of weekly PCR testing.^28^

# Data resources

- 1. **Testing data**

We obtained separate data files from the United Kingdom Health Security Agency (UKHSA) for LFD and PCR testing data through the Pillar 2 testing programme (swab testing for the virus in the wider population, through commercial partnerships, either processed in a laboratory or more rapidly via LFD tests). These data were then filtered such that “channel=Care Homes” and “UseCase=ADULT_SOCIAL_CARE”, with “OrganisationRole” either “resident” or “staff”. For each of these datasets, we aggregated testing data by week, “siteid”, “SiteName”, and “OrganisationRole”.

- 1. **Death data**

Data for COVID-19-related deaths in care home residents were supplied by CQC. The date of each death was the date the death was notified to CQC; notifications about deaths must be sent directly to CQC without delay (and CQC holds that notifications are typically provided within 2–3 days of death). The “type of death” included in these data was either “confirmed” or “suspected”, and for the majority of analyses, these two categories were aggregated into a “total COVID-19-related deaths” measure. These data were by week at the individual care home level and covered the period 1 April 2020–31 August 2022, but only data within our analysis period (October 2020–March 2022) were used in this study. We had neither data on staff mortality due to COVID-19 nor information of locations where the deaths occurred.

- 1. **Care home characteristics**

Data on individual care home characteristics were obtained from files provided by UKHSA and the NHS NECS Capacity Tracker data, also provided by UKHSA. These data included information on total resident count, total care worker count, the lower tier local authority (LTLA) within which each care home is located, the latest CQC rating of each care home (a static measure as CQC ratings were suspended during the pandemic), the primary client type served by the care home (e.g. older people (≥65 years) or those with learning disabilities), whether the care home was a nursing home, whether it was an acute care home, whether it was a community care home, and measures of care-home COVID-19 response (e.g. whether visiting was allowed).

- 1. **COVID-19 community prevalence**

To determine COVID-19 prevalence, we used data from REACT, a randomised surveillance study conducted in the UK.^29^ The REACT data consisted of numbers of tests conducted by week within each LTLA, along with the results of these tests. In week-LTLAs with fewer than five observations, the data were censored if there were any positives to ensure the data were not identifiable. As the number of PCR tests conducted through the REACT programme in each LTLA in a given week was relatively low, this limited its direct usefulness in our statistical models. Instead, we used the methods described by Bajaj et al.^30^ to obtain higher precision estimates of weekly LTLA-level COVID-19 prevalence by leveraging the Pillar 2 PCR testing data, which had much greater testing volumes. Specifically, this method uses the prevalence data collected within larger scale, “coarse” regions to understand biases in mass testing data collected within the same regions. The models fitted to these coarse regions are then used to estimate prevalence at a finer scale using only the mass testing data. By so doing, this method assumes that the biases in test seeking behaviour inherent within the coarse regions are the same as in the fine-scale ones. Here, we followed Bajaj et al.^30^ by choosing the 9 PHE regions of England to be the coarse regions and the 321 LTLAs in England to be the fine-scale regions. We fit the model to the PCR testing data and REACT data from 25 July 2020 to 2 April 2022. The measure of COVID-19 prevalence for each care home used within our models corresponded to the posterior mean prevalence estimated by the above method corresponding to the LTLA encompassing each care home.

- 1. **SARS-CoV-2 lineages**

Our weekly data on counts of SARS-CoV-2 lineages by week and LTLA were obtained from the Sanger Institute website.^31^ These counts were then binned according to whether they corresponded to the Alpha, Delta, or Omicron variants of concern (VOCs), or whether they were from “other” lineages, and these data were used to determine weekly LTLA-level proportions of each of these VOCs.

- 1. **Vaccination**

Our care homes vaccinations data were obtained from UKHSA, and each row of this dataset corresponded to a vaccination carried out on a particular individual (anonymised through a pseudo-identifier), including information of the dose type (dose 1, dose 2, dose 3, booster, spring 22 booster, or null) and the role of the individual within the organisation (e.g. whether an individual was resident or staff). As we lacked individual-specific care home composition data, our measure of weekly vaccination coverage was approximate and consisted of the total counts of individuals with a particular role (here, residents or staff) vaccinated within a care home divided by the population size of that group. This measure likely overstates vaccine coverage, since vaccination doses from individuals who exited care homes within the study period would continue to contribute to coverage. But, because many care homes comprised vulnerable populations, it is likely that their vaccine coverages were nonetheless high.

- 1. **Regional population** **structure**

In a number of our analyses, we required population sizes of regions within England. We used the 2020 mid-year estimates of population sizes produced by the Office for National Statistics (ONS), which were publicly available and obtained from the ONS website.

- 1. **Testing policy changes**

We included four variables that aimed to capture the impact of key policy shifts for adult social care staff testing (as communicated by UKHSA) on reported staff testing intensity. The variables included in our regressions to represent each of these four policy shifts were binary indicator variables: equal to 0 before the date of the change and equal to 1 afterwards. These indicators are useful for capturing rapid shifts in staff testing occurring immediately following policy updates, but less so if the effects of the policy change were realised more gradually. We included only the 13 April 2021 policy variable in our staff PCR regression, as this was the only policy change that affected staff PCR testing, and our models were unable to attribute changes in testing intensity with the advent of this policy.

Specifically, these were the following, as shown in Table S1:

- 23 December 2020 – introduction of LFD testing in care homes: twice-weekly asymptomatic testing for staff and for all visitors and visiting professionals.
- 13 April 2021 – after the expansion of regular asymptomatic testing with PCR and/or LFD depending on the sector during the spring of 2021, by 13 April 2021 every social care worker in England had access to regular asymptomatic testing.
- 15 December 2021 – intensification from twice-weekly LFD testing to thrice-weekly LFD testing for staff in care homes and in high-risk extra care and supported living and day care centres in response to the threat posed by the Omicron variant over the winter.
- 16 February 2022 – intensification from thrice-weekly to daily LFD testing (also known as “testing before starting work each day” or “pre-shift”), in response to the threat posed by peaks in the Omicron variant over the winter.

1. **Data cleaning**

## Interpolation of time-varying care home data

We obtained care home-specific, time-varying covariate data from the NHS NECS Capacity Tracker. For each covariate of interest, we extracted weekly time series data from the capacity tracker data file, using the “weekly_last_updated” column as the time value; when a single week had multiple observations, we took the mean of the observations. We extrapolated the first and last observations in the time series to the beginning and end of the evaluation period, assuming that the value of the covariate remained constant before the first observation and after the last observation. For care homes with data missing in certain weeks, we used linear interpolation to obtain values for the covariate for the missing weeks. Using this approach, we considered the following variables from the NECS Capacity Tracker file: “total_resident_count”, “care_workers_employed”, “care_workers_absent”, “agency_care_workers_employed”, “aprons_pressure”, “masks_pressure”, and “is_visiting_allowed”.

## Vaccination level

To approximate the level of vaccination in a care home’s residents or staff members, we aggregated the total number of doses administered at a particular care home (dose 1, dose 2, dose 3, booster, and spring 2022), by staff or resident, and divided this by the number of staff members (care_workers_employed) or residents (total_resident_count) in particular weeks. This measure was a relatively crude metric of vaccination coverage, as it failed to account for any turnover in the care home memberships over time. However, we lacked data on individual care home memberships, so our measure likely overstated the level of vaccination coverage, particularly so for those care homes with a high turnover of residents or staff.

## Merging of testing data and deaths data

We obtained COVID-19 testing data from UKHSA, in which individual tests and their results were listed. Each test was associated to a particular care home by its Site ID (siteid) and Site Name (sitename). We also obtained data from the CQC for the number of weekly COVID-19-related deaths per care home reported to the CQC, with each care home indicated by its Location ID and Location Name. (Some care homes appeared in our reference set but had zero deaths and thus did not appear in our deaths data; however, for these care homes we also had Location ID and Location Name information from the CQC Care Directory with filters.) Because a care home’s Site ID (testing data) and Location ID (deaths data) are different identifiers, we merged testing and deaths data using the dim_satellite_organisations file (obtained from UKHSA), which contains for each care home a legacy_carehome_id (equivalent to Location ID from deaths data) and an organisation_id (equivalent to siteid from testing data).

## Merging of testing/deaths data with other covariates

We merged time-varying data from the NHS NECS Capacity Tracker using the cqc_id (equivalent to Location ID). We merged non-time-varying care home metadata from NECS using the cqc_id (equivalent to Location ID). We merged vaccination data using the OrganisationId (equivalent to Site ID).

# The association between care home characteristics and reported testing intensity of LFD and PCR tests among residents and staff

In individual care homes, the reported PCR testing intensity represented a strong monthly periodicity among residents but was generally more stable and consistent among staff (Figure S1 and Figure S2). For LFDs, the reported testing intensity was relatively stable, albeit with step changes in testing among both residents and staff.

To examine further the factors associated with changes in test reporting intensity, we performed a series of regression analyses as shown in Table S4: one regression for each of resident PCR, resident LFD, staff PCR, and staff LFD. These regressions contained a range of time-varying (e.g. past test intensities and positivity rates) and time-invariant characteristics (e.g. CQC rating and whether a care home was a nursing home). These models were linear regressions with dependent variables given by the corresponding reported testing intensity in a week in a given care home.

The *R*^2^ statistic was considerably lower for the resident PCR regression than for the LFD equivalent, with only around 16% of overall variation in reporting intensity explained by the model versus around 39% for the LFD model. This is largely illustrative of the more idiosyncratic test reporting for resident PCR testing that occurred at the care home level and indicates that LFD testing intensity was more predictable (from the specified regression). The mean reported test intensities across the evaluation period were approximately 0.26 reported tests per resident for PCR and 0.06 per resident for LFD tests (both of these are weekly measures).

The results presented in the main text “outbreak testing” showed the difficulty in determining how changes in testing affected cases, because naïve regressions of positive tests on reported test intensities uncovered a positive association due to the strong response of test intensities to the numbers of positives uncovered (see Table S5).

For LFD and PCR tests among staff, the testing intensity exhibited strong positive autocorrelation with the previous weeks’ test intensities. Also, for both test types, the models were substantially better able to explain the variation in test intensities versus the resident regressions, and the R^2^ was more than 0.6 for both regressions.

Counterintuitively, for both regressions, higher average numbers of positive results per care home member were associated with decreases in reported test intensity in the following week; it is unclear what mechanism drove this association.

# The association between outbreak size when they were initially uncovered and care home characteristics in outbreak discovery

We investigated how testing intensity in both residents and staff influenced the number of positive tests found in the following week for care homes not in outbreaks in the first week, but which uncovered positives in the following week. We modelled this process using Equation (2), shown in the main text.

A number of assumptions are inherent here: when the local prevalence is zero, there can be no positives; this would be violated should visitors, residents, or staff enter the care home from outside the LTLA; it would also be violated due to the presence of false-positive test results (most relevant for LFD tests).

Specifying a Poisson likelihood is a strong assumption: this was chosen because, while negative binomial regressions fit the data better, these models often suffered convergence issues and were numerically unstable. When the negative binomial regressions did converge, however, the odds ratios associated with testing were similar to those of the Poisson model. The above model does not include random intercepts for the individual care homes, as models doing so failed to converge. The regression results for this model are shown in Table 2 in the main text. Models incorporating diminishing returns from testing provided a more predictive fit to the data than those without it, so, in the results we present, we transformed these variables to account for this.

We presented some results in the main text and stated that “staff testing in the previous week was associated with smaller outbreaks in either residents or staff when they were initially uncovered – in other words, the outbreaks were detected earlier”. Testing in residents was not as strongly associated with the average initial outbreak size in residents and, for staff, had no association with the outbreak”. This broadly held across analyses involving different subsets of regressors (columns (1) and (2) in Table S6 and Table S7). We also performed additional analyses where the test intensities were broken down into resident PCR, resident LFD, staff PCR, and staff LFD (columns (3) and (4) in Table S6 and Table S7). The initial outbreak size in residents was more strongly negatively associated with testing residents in the previous week via LFD than PCR (columns (3) and (4) in Table S6). The initial outbreak size in residents was also negatively associated with staff testing, either via LFD or PCR, with the magnitudes of these effect sizes similar across both test types (columns (3) and (4) in Table S6). The association between the initial outbreak size in staff and either type of resident testing was weak and sometimes of conflicting signs dependent on the regression specification (columns (3) and (4) in Table S7).

The COVID-19 prevalence coefficients are omitted in Table 2 in the main text to aid readability and indicated a strong negative association between testing intensity and prevalence. This reflects that there was a nonlinear association between prevalence and testing intensity (as this variable also appeared as an offset), where increases in prevalence have declining impacts as prevalence increases.

The effect sizes associated with the vaccination variables were relatively small and may reflect the relatively imprecise measures to which we had access.

By conducting an ANOVA on the deviance scores of the regressions shown in Table 2 in the main text, we were able to approximate the relative contributions of each variable to the overall predictive accuracy of the models, as each variable is added to the regression in turn. These results are shown in Table S8 (for resident outbreak discovery) and Table S9 (for staff). These indicate the importance of the testing variables, particularly that staff testing is a strong predictor of outbreak size in the following week. They indicate that CQC ratings have only a moderate impact on the model’s predictive power. Including whether a care home was a nursing home in the regression led to large improvements in predictive accuracy, as did accounting for the primary type of client each care home services. Accounting for the local level of the Omicron variant and the size of care homes also substantially improved the model predictions, as did accounting for the diminishing impact of increases in prevalence on initial outbreak size. Including the measure of care home vaccination intensity substantially improved the predictive accuracy, but the results in Table 2 in the main text show that these variables have small effect sizes. This is possibly because these vaccination variables were imprecise and mirrored nationwide time trends and, once the weekly time variables were included in the regression, these variables had minimal predictive power.

In Tables S10 (residents) and S11 (staff), we show sensitivity analyses where we perform the same regressions as in Table 2 in the main text, for three distinct time periods:

- 1 October 2020 to 31 December 2020, broadly taken to be prior to substantial vaccine-induced immunity
- 1 January 2021 to 30 November 2021, the pre-Omicron phase
- 1 December 2021 to 31 March 2022, the Omicron phase

Across all three time periods and across the resident and staff regressions, the association between staff testing and the size of outbreaks when discovered was of consistent sign and of large magnitude. In the staff regression, the association became more pronounced over time. This contrasted with the coefficient for resident testing, whose sign fluctuated for the resident outbreak regressions, and which only became negatively associated with outbreak size during the Omicron phase.

Although the association of CQC rating with outbreak size varied across the time periods and resident and staff regressions, those care homes rated as outstanding consistently had smaller outbreaks (when initially discovered) versus the other cohorts. Generally, the association of CQC rating was greatest in the pre-Omicron phase.

In both the resident and staff regressions (Table S10 and Table S11), the association between initial outbreak size and the primary type of client served by the care homes varied throughout the evaluation period, but the signs of effects were maintained throughout. Of particular note, the effect sizes associated with care homes serving primarily older persons (aged ≥65 years) or individuals with dementia declined in the Omicron phase. The association of the fraction of agency workers with initial outbreak size changed sign throughout the evaluation period: in the first period, it was associated with smaller outbreak sizes; in later periods, it was associated with larger ones, across both residents and staff.

# The association between subsequent outbreak sizes during outbreaks and care home characteristics in outbreak control

We investigated how, subsequent to positive cases being found within a care home, the response was able to identify (and presumably isolate) cases, leading to reductions in the size of the outbreaks in subsequent weeks. To do so, we considered only weeks where the previous week had at least one positive case in either staff or residents. We modelled the process using Equation (3), shown in the main text.

An assumption here is that, during an outbreak, new cases arise predominantly from previous ones within the care home. While it is possible that, during an outbreak, additional cases could be imported from outside a care home, these introductions may be relatively rare, and we assume that the majority of new cases are due to those occurring in previous weeks. We do, however, allow for importations, through a prevalence term included in the additional covariates.

The results of this regression for positive test counts in residents and staff are shown in Table 2 in the main text. We found a strong association between past testing intensity and positive counts, where higher levels of past testing were associated with fewer positives. This effect was particularly strong for past staff testing on staff outbreaks and for past resident testing on resident outbreaks – again, this supports the hypothesis that these groups tended to associate more with themselves as opposed to intergroup mixing.

If a substantial proportion of individuals in a care home are infected in a given week, the number of positives in the following weeks could be low because there were few susceptible individuals left to infect. If large outbreaks were accompanied by high levels of testing, this would then make it appear (falsely) as if a high testing intensity drove down infections. To investigate this hypothesis, we performed an additional regression where we included the average number of infections per care home member in the previous week (Table S12). In both resident and staff regressions, this regressor was significant and negatively associated with outbreak size. As this regressor also appears in the offset term, the negative association does not indicate a negative impact of tests in the previous week on those in the current week but indicates that there were diminishing returns to the impact of positives on future transmission. One possible mechanism for these diminishing returns could be, as discussed, the depletion of susceptible individuals. The impact of incorporating this additional regressor was to reduce the effect size associated with testing, but the effects remained of the same sign and the general trends were the same (e.g. that testing in residents had the largest negative association with future outbreak size in residents and that testing in staff had the largest negative association with future outbreaks in staff).

Increases in the LTLA-level prevalence were associated with increased positives (this estimate is omitted from the regression table for readability), presumably through further introductions of cases into care homes from either the most likely route, e.g. staff, visiting professionals, or visitors, or potentially through new admissions or residents returning from being outside of the care home for a period of time. The effect size here was large but reflected the scale of prevalence (0–1) and that, typically, prevalence was low (typically less than 0.01).

In Table S13 and Table S14, we investigate the stability of key regression coefficients by time-period, considering the same three periods as in Table S6 and Table S7.

In all time periods, the effect size of testing in residents was of the same sign and relatively stable – this was true for both the resident regression (Table S13) and staff regression (Table S14). This was also the case for the effect size associated with testing in staff, although in the staff regressions, the association between testing in staff and outbreak size became more pronounced over the course of the evaluation period. Across both regressions, there was heterogeneity in the association of CQC rating with outbreak size. In the first period, the “inadequate” care homes fared relatively well in the resident regression, with minimal differences in the staff regression. In the pre-Omicron period, the order was the same as in our main regression, with “outstanding” care homes faring best. In the final period, there was little association across CQC ratings and outbreak size. Across all periods, the effect size associated with serving primarily older persons or those with dementia was positive and of similar size; for both of these factors, the effect sizes were greater in the first period and greater for the resident regressions. The association between the number of care workers per resident and outbreak size was of the same sign (negative) across each of the time periods and relatively stable.

# The association between COVID-19-related mortality and care home characteristics

By using the design illustrated in Figure 1, we could, in principle, miss deaths from positives that occurred more than 2 weeks after the positive was reported (e.g. the blue block in Figure 1 in the main text). We could also misattribute deaths to a particular block if the delay from testing positive to death was either short or long. However, we view this as a largely unavoidable smoothing of the data, which would remain for any other choice of block size. An improved analysis would consider individual-level paired testing and deaths data (to which we did not have access).

# Deaths averted under hypothetical testing scenarios

We used the fitted regression models describing outbreak discovery and outbreak control together with the model for COVID-19-related deaths to estimate the number of such deaths that would occur under counterfactual testing and care worker scenarios.

Inherently, our approach is statistical opposed to being mechanism-based and, because of this, a number of additional assumptions were required to produce reasonable projections. A key assumption of all of our projections is that our regression model estimates represent causal effects, which is unlikely to be true and suggests caution is needed in interpreting our results.

We provide measures of uncertainty in our projections, which are solely based on the uncertainty in the negative binomial regressions that link positive test results with deaths. This, however, likely understates the true uncertainty in the projections, as it fails to account for uncertainty in the structure of the models. It also fails to account for the inherent uncertainty in epidemic dynamics, which is particularly acute in care homes, where the relatively few individuals in each care home means that the individual outbreaks in each care home would unfold in a relatively unpredictable manner.

# Deaths averted across testing scenarios

We performed a sensitivity analysis where we used the outbreak models, which allowed for nonlinear effects of past positives (e.g. if there was depletion of susceptible individuals). These models estimated smaller but still marked effects of testing on COVID-19-related deaths (Figure S4). Under a 50% reduction in testing, this model predicted that deaths would increase by 27,660 (uncertainty interval (UI), 23,000–32,900), a 111% increase. For a 25% reduction, deaths would increase by 40% (UI, 27%–55%). For a 25% increase in testing, this projection indicated that 15% of deaths would have been averted (UI, 5%–24%); and for a 50% increase, 28% of deaths would have been averted (UI, 19%–36%).

# Deaths averted after increasing staff numbers scenarios

We also considered how increases in the number of staff could have influenced COVID-19-related deaths in care homes within adult social care. In Figure S5, we show the results of a sensitivity analysis where we used the outbreak models, allowing for nonlinear relationships with past positives (Table S12). These projections estimated a slightly smaller influence of changes to the number of staff per resident on COVID-19-related deaths. A 25% increase in staff per resident would reduce deaths by 8% (UI, -3% to 17%) and a 50% increase would reduce deaths by 17% (UI, 7%–26%).

# Test volumes, costs, and cost effectiveness

### Data processing

Both cost and volume data were provided by UKHSA. We used the Cost Allocation Project (unpublished internal project) conducted by UKHSA for the ONS as a starting point for our analysis. Testing cost data were primarily sourced from UKHSA general ledgers. Where funds were held by another department (e.g. DHSC), these had either been transferred or were added as an adjustment to the values at the point of analysis. The line items in the cost data we received were not directly mapped to the focus of our analysis (e.g. care homes). Therefore, the costs used throughout this analysis are estimates based on adjustments we made. We apportioned all costs to the various testing services (e.g. adult social care) for each technology based on the applicable service volumes. Test volume data (purchased and distributed) were provided by UKHSA. All costs and volumes used in the evaluation are for England only. We removed line items for the devolved administrations (DAs) before analysis. The DAs constituted approximately 12% of test volumes for financial year (FY)21 and 16% for FY22. We calculated unit costs for the full evaluation period, which captured the purchase price of tests, as well as all other direct, indirect, and overhead costs associated with the testing programme. These included the logistics, human resources, and other costs to deliver a test to the point of care. Unit costs exclude support payment costs and laboratory set-up costs. As unit costs decreased over time, the average cost differs by financial year.

### Cost-effectiveness analysis

Table S15 summarises the input parameters and sources used for the economic evaluation. We conducted a sensitivity analysis that tested the sensitivity of the outcome to the quality-adjusted life-years (QALYs) for death (presented in Figure 3) with a minimum and maximum value of QALY for deaths of 4.98 and 8.8, respectively. The full set of estimates for the cost-effectiveness analysis are presented in Table S16.

# Study limitations

Predicting COVID-19-related deaths in care home residents under counterfactual responses is inherently complex. The thousands of individual care homes are generally small, and the outbreaks thus start and evolve in each of them in highly unpredictable ways. Care homes are also diverse, with the responses they made and their characteristics and dynamics imperfectly measured. Our analysis represents a first step towards estimating the impact of testing on COVID-19-related deaths in care home residents, but it has a number of important limitations, which we discuss in this section.

We used the fitted regression models describing outbreak discovery and outbreak control together with the model for COVID-19-related deaths in residents to estimate the number of such deaths that would have occurred under counterfactual testing and staff scenarios. However, because we did not have a mechanistic model that linked outbreak response to changes in the size of epidemics (and the number of positive test results), we were required to make a number of additional assumptions. Future work should consider the use of more epidemiologically informed, mechanistic, individual-based models that describe the evolution of outbreaks in care homes and how changes to testing or the number of workers may affect their course. The regression model results that we present here should be useful in parameterising such models.

One key characteristic of outbreaks in care homes is that there is a small number of individuals who could become infected; this number initially diminishes and later increases as individual-level immunity is gained then subsequently wanes following recovery from infection. We attempted to proxy for the depletion of susceptible individuals in our regression models by allowing non-monotonic impacts of the previous week’s positives on subsequent infections: models including these effects did predict fewer deaths averted due to testing, but the differences in effect sizes were modest. This may be because the typical number of positive tests occurring per week (if positive tests were recorded) was generally small compared with the overall care home size. It may also be because we made additional assumptions when projecting deaths, which aimed to account for the depletion of susceptible individuals. An individual-level mathematical model that considered individuals’ dynamic shifts in immunity (following either natural infection or vaccination) would be a useful tool to further probe how changes to outbreak responses would affect deaths.

A crucial assumption we make when predicting how changes to testing or numbers of care home workers would affect COVID-19-related deaths is that our regression results represent causal effects. While we have attempted to control for important confounders, there is a range of factors that we could not accurately measure (such as individual immunity – our measure of vaccine coverage was imprecise as we did not have access to individual-level care home memberships) and thus could not precisely model. Additionally, while our models may reflect reasonable associations across all care homes on average, it is likely that these associations would vary idiosyncratically across individual care homes. The models we fitted did not generally include such effect modification at the care-home level, either because the models incorporating such random effects did not converge (likely due to the often-limited number of observations at the care home level) or because the models could not be fitted owing to the extensive computational resources required to fit these models to these large datasets. Because of this, our predictions may mischaracterise counterfactual considerations of the impact of false-positives. Particularly during time periods when there was low transmission, it is likely that a number of isolated occurrences of positive tests represented not outbreaks but false-positive results (particularly if the tests were conducted using LFDs). These faux outbreaks would not evolve in the same way as real ones, and the outbreak responses could thus be wasteful. However, because full outbreak responses required a number of positive test results in order to be enacted, these occurrences may have been relatively rare. Our models did not allow for a different response of COVID-19-related deaths in relation to false-positives versus true-positives, therefore it is possible that, particularly during low transmission periods, our statistical models overstated the influence of testing on deaths.

However, as these were periods defined by relatively few cases (and COVID-19-related deaths) and the model predictions were similarly so (see Figure 4), it is unlikely that this effect would lead the model predictions to strongly overstate the number of deaths.

Testing without accompanying changes in behaviour or actions following positive test results, for example through the isolation of positive cases, cannot affect COVID-19 outcomes. Our projections implicitly assume that these accompanying effects remain in place under changes to the level of testing. We performed analyses on subdivisions of the evaluation period (Tables S12, S13, S15, and S16) that did show some evidence of variation in the effect size associated with testing, which may be due to changes in behaviour of staff/residents following positive test results.


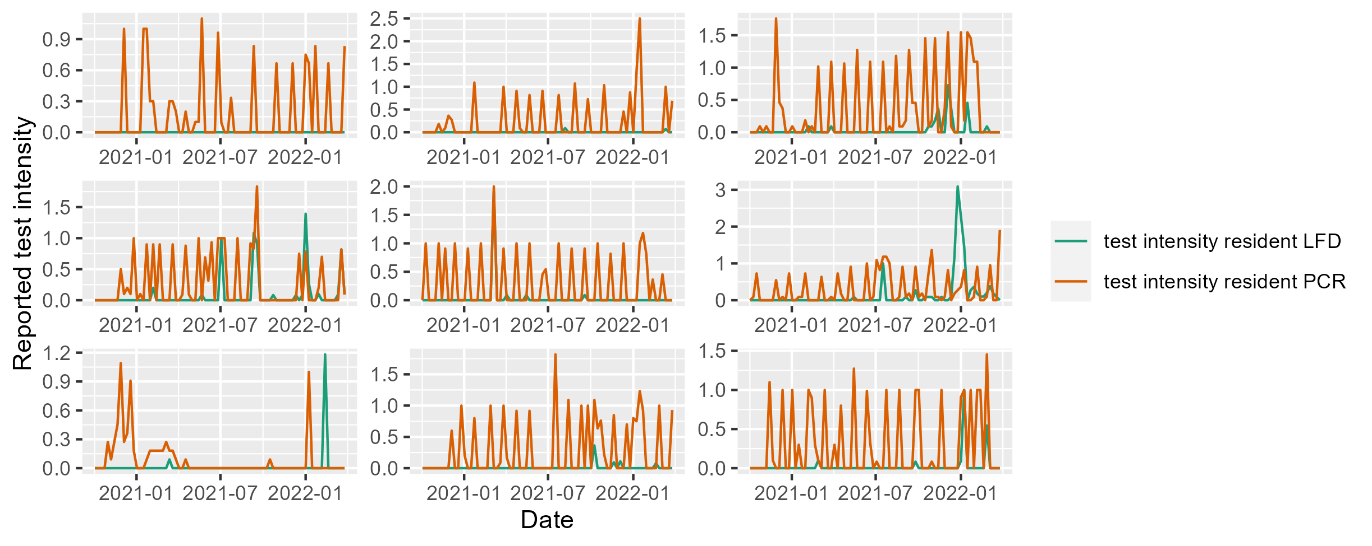


# Figure S1. Example resident reported test intensity with LFDs and PCR in small care homes.

These nine care homes were the 9000^th^–9008^th^ largest care homes (of those with more than ten residents) according to mean total resident count.


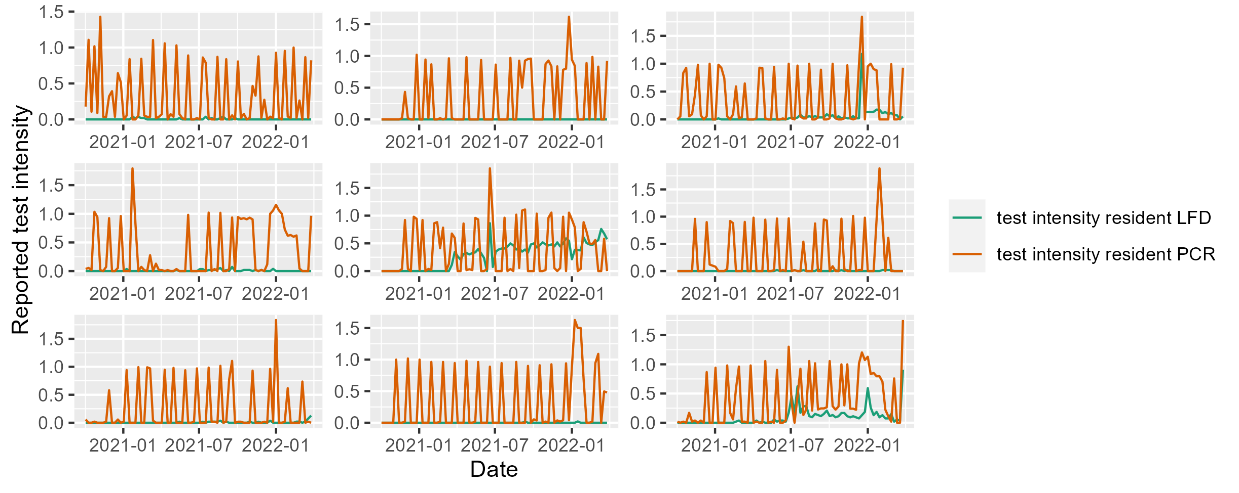


# Figure S2. Example resident reported test intensity with LFDs and PCR in mid-sized care homes.

These nine care homes were the 1000th–1008th largest care homes (of those with more than ten residents) according to mean total resident count.


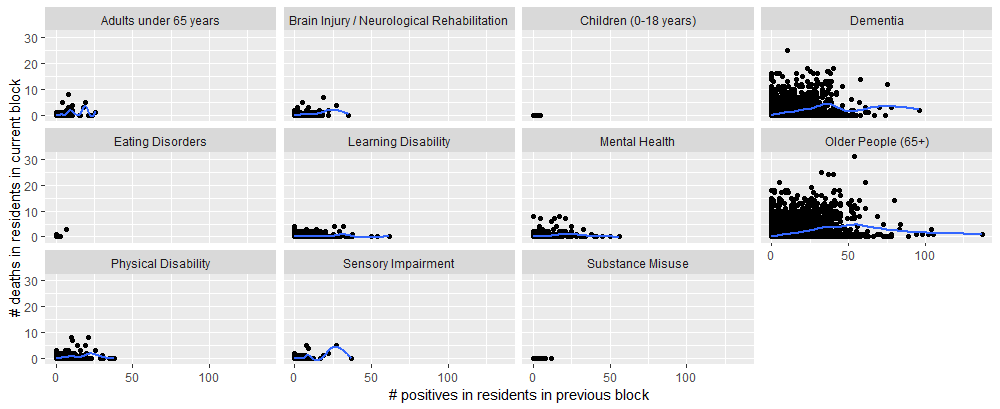


# Figure S3. Associations between the number of positives and the number of COVID-19-related total deaths, by primary client type served by care homes.

Considering the complexity of the time-dependent regression analyses, we only focused on care homes whose primary clients are labelled as ‘older individuals (>65 years)’, ‘individuals with dementia’ and ‘individuals with learning disabilities’ which account for 47.4%, 12.7% and 28.6%, in total 88.7% of the total number of care homes in the main text. In this plot, the points indicate block-level observations for a particular care home; the blue lines represent linear regression fits assuming a generalised additive model (the default chosen by ggplot2) between numbers of positives and numbers of deaths.


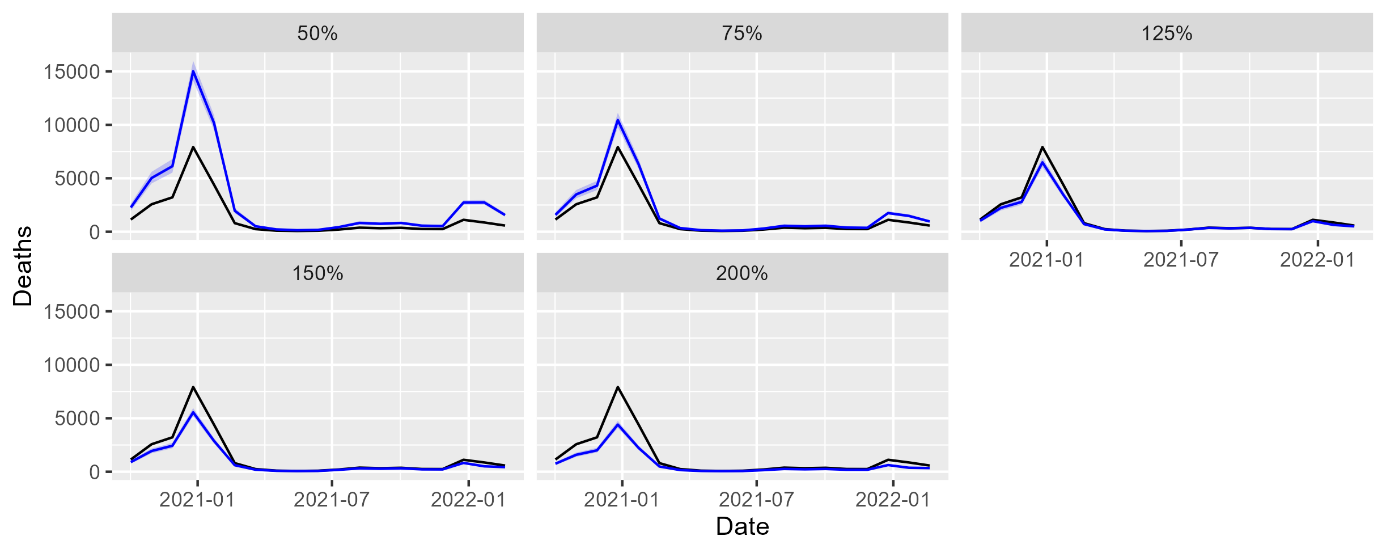


# Figure S4. Projected COVID-19-related total deaths under counterfactual testing scenarios: sensitivity analysis.

Each plot shows the actual deaths (black lines) and the projected lines (blue lines) with associated uncertainty (see Methods). Each panel corresponds to a different counterfactual testing scenario when the numbers of tests were at the levels shown at the top of each panel relative to the historical levels: e.g. 75% means that testing (in both residents and staff) was at 75% of its factual level. This projection was made using the outbreak models incorporating diminishing returns to lagged positives (shown in Table S11).


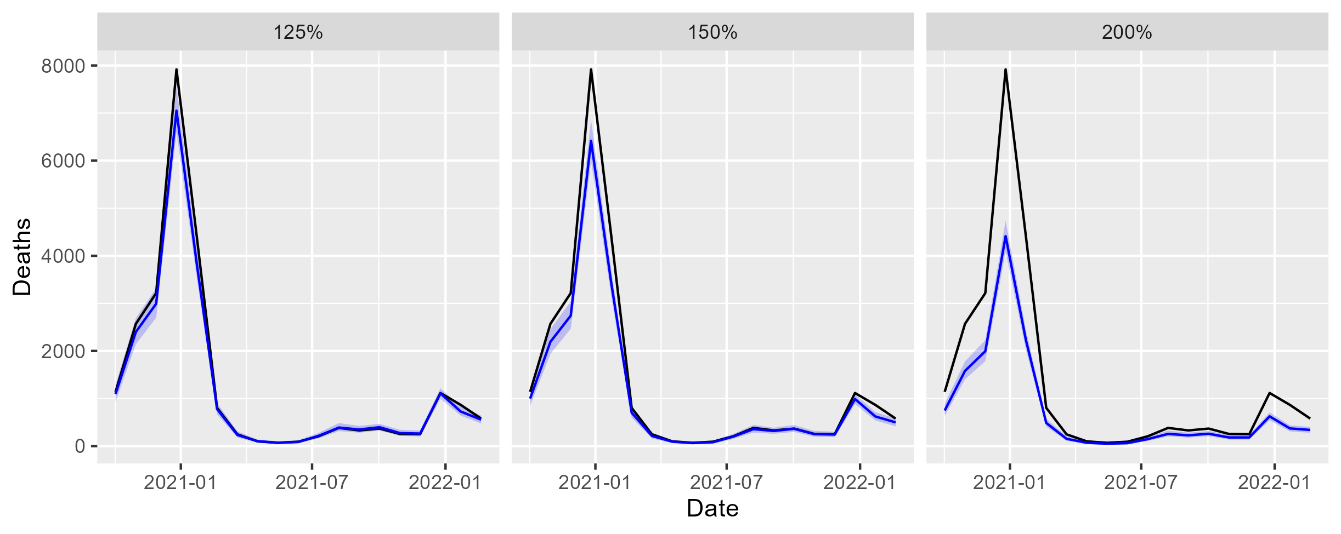


# Figure S5. Projected COVID-19-related total deaths under counterfactual staff scenarios: sensitivity analysis.

Each plot shows the actual deaths (black lines) and the projected lines (blue lines) with associated uncertainty (see Methods). Each panel corresponds to a different counterfactual testing scenario when the numbers of staff per resident were at the levels shown at the top of each panel relative to the historical levels: e.g. 125% means that the number of staff per resident was increased by 25% from its factual level. This projection was made using the outbreak models incorporating diminishing returns to lagged positives (shown in Table S11).

# Table S1. Description of testing policy in care homes in England.

| **Testing target** | **Testing type** | **Policy time-period** | **Intended testing intensity by the policy** |
| --- | --- | --- | --- |
| Staff | PCR | October 2020– February 2022 | One test per week per staff member^25^ |
|  |  | 16 February 2022–March 2022 | Removal of weekly PCR testing^32^ |
|  | LFD | December 2020–December 2021 | Twice-weekly LFD testing^22 32^ |
|  |  | December 2021–March 2022 | LFD testing three times a week^25 26^ |
| Resident | PCR | October 2020–March 2022 | Regular testing: one test per month per resident  Outbreak testing: day 1, 4, and 7 testing |
|  | LFD | February 2021, outbreak testing | Outbreak testing: day 1, 4, and 7 testing^23^ |

# Table S2. Number of LFDs distributed and PCR tests registered in England as part of the adult social care testing service (during the evaluation period).

Note that volumes have been rounded to the nearest hundred.

| **Service** | **Description** | **Total number of tests** | **Percentage of total** |
| --- | --- | --- | --- |
| Adult social care  (all services) | Number of LFD tests distributed | 227,317,900 | 11.4% (of all LFDs distributed in England) |
|  | Number of PCR tests registered | 40,807,000 | 35.5% (of all PCR tests registered in England under Pillar 2) |
| Care homes only | Number of LFD tests distributed | 189,541,200 | 9.5% (of all LFDs distributed in England) |
|  | Number of PCR tests registered | 33,056,800 | 28.7% (of all PCR tests registered in England under Pillar 2) |

# Table S3. Total financial cost of the adult social care testing service for England (during the evaluation period).

Note that costs have been rounded to the nearest hundred.

| **Service** | **Test type** | **Total cost (GBP)*** | **Percentage of total spend (England)**** |
| --- | --- | --- | --- |
| Adult social care (all) | LFD | 1,523,531,300 | 6.5% |
|  | PCR | 3,298,160,200 | 14.1% |
|  | Total | 4,821,691,500 | 20.6% |
| Adult social care (care homes only) | LFD | 1,208,877,200 | 5.2% |

**Table S4. Regression results for reported testing intensities (PCR and LFD tests) among residents and staff.** All sets of regression results represent estimates for linear models fitted using ordinary least squares. The figures in parentheses represent 95% confidence intervals for the coefficient estimates.

|  |  | | **Number (%) of care homes** | **Resident PCR testing intensity** | **Resident LFD testing intensity** | **Staff PCR testing intensity** | **Staff LFD testing intensity** |
| --- | --- | --- | --- | --- | --- | --- | --- |
| Outbreak size | Average number of positive results per care home member | | / | 0·293^***^  (0·273, 0·314) | 0·141^***^  (0·128, 0·153) | -1·893^***^  (-1·946, -1·840) | -0·153^***^  (-0·226, -0·079) |
| CQC rating | Inadequate (baseline) | | 302 (2·0%) |  |  |  |  |
|  | Requires improvement | | 2,421 (16·4%) | 0·013^***^  (0·006, 0·020) | 0·003  (-0·001, 0·008) | 0·016^*^  (-0·003, 0·034) | 0·038^***^  (0·012, 0·063) |
|  | Good | | 11,197 (75·6%) | 0·017^***^  (0·010, 0·023) | 0·007^***^  (0·002, 0·011) | 0·028^***^  (0·010, 0·046) | 0·060^***^  (0·035, 0·084) |
|  | Outstanding | | 634 (4·3%) | 0·030^***^  (0·022, 0·038) | 0·015^***^  (0·010, 0·020) | 0·042^***^  (0·021, 0·063) | 0·100^***^  (0·071, 0·130) |
|  | No record | | 251 (1·7) | 0·009  (-0·007, 0·025) | 0·001  (-0·009, 0·011) | 0·035^*^  (-0·007, 0·076) | 0·044  (-0·014, 0·101) |
| Primary clients | Older individuals (≥65 years) | | 7,020 (47·4%) | 0·005^***^  (0·002, 0·008) | -0·010^***^  (-0·013, -0·008) | 0·004  (-0·005, 0·012) | 0·020^***^  (0·008, 0·032) |
|  | Individuals with dementia | | 1,881 (12·7%) | 0·002  (-0·002, 0·007) | -0·010^***^  (-0·013, -0·008) | 0·004  (-0·007, 0·015) | 0·015^**^  (0·0003, 0·030) |
|  | Individuals with learning disabilities | | 4,233 (28·6%) | -0·030^***^  (-0·033, -0·026) | 0·005^***^  (0·003, 0·007) | 0·013^***^  (0·004, 0·022) | 0·039^***^  (0·027, 0·052) |
| Local proportion of VOC | Alpha (baseline) | |  |  |  |  |  |
|  | Delta | | / | 0·025^**^  (0·001, 0·050) | -0·008  (-0·024, 0·007) | -0·043  (-0·106, 0·021) | 0·001  (-0·088, 0·089) |
|  | Omicron | | / | 0·010  (-0·012, 0·031) | -0·001  (-0·015, 0·012) | 0·035  (-0·021, 0·091) | -0·002  (-0·080, 0·076) |
| Care home size | Number of care workers per resident (staff-to-resident ratio) | | / | 0·010^***^  (0·009, 0·012) | -0·001^*^  (-0·002, 0·0001) | -0·029^***^  (-0·034, -0·024) | -0·037^***^  (-0·044, -0·030) |
|  | Fraction of agency workers | | / | -0·015^***^  (-0·020, -0·011) | -0·001  (-0·004, 0·002) | 0·030^***^  (0·018, 0·042) | 0·022^**^  (0·005, 0·038) |
|  | Total resident count | | / | 0·0004^***^  (0·0003, 0·0005) | -0·0001^***^  (-0·0002, -0·0001) | -0·001^***^  (-0·001, -0.0004) | -0·001^***^  (-0·001, -0·0005) |
| Care home type | Community care home | | 522 (3·5%) | 0·004  (-0·001, 0·009) | 0·005^***^  (0·001, 0·008) | 0·006  (-0·008, 0·019) | -0·007  (-0·025, 0·012) |
|  | Acute care home | | 2 (0·01%) | -0·001  (-0·079, 0·077) | -0·019  (-0·067, 0·030) | -0·109  (-0·311, 0·092) | -0·178  (-0·458, 0·102) |
|  | Nursing home | | 4,176 (28·2%) | 0·003^**^  (0·0003, 0·005) | -0·002^***^  (-0·004, -0·001) | 0·023^***^  (0·016, 0·029) | 0·015^***^  (0·006, 0·024) |
|  | Independent organisation | | 10 (0·07%) | -0·081^***^  (-0·118, -0·044) | -0·018  (-0·042, 0·005) | 0·208^***^  (0·112, 0·303) | 0·399^***^  (0·267, 0·532) |
| COVID-19 in the community | Community infection prevalence | | / | 0·900^***^  (0·676, 1·125) | -0·136^*^  (-0·276, 0·004) | -0·213  (-0·793, 0·367) | 1·132^***^  (0·325, 1·939) |
|  | Local proportion of variant of concern (baseline: Alpha) | Delta | / | 0·025^**^  (0·001, 0·050) | -0·008  (-0·024, 0·007) | -0·043  (-0·106, 0·021) | 0·001  (-0·088, 0·089) |
|  |  | Omicron | / | 0·010  (-0·012, 0·031) | -0·001  (-0·015, 0·012) | 0·035  (-0·021, 0·091) | -0·002  (-0·080, 0·076) |
| Testing policy changes | Twice-weekly LFD testing in staff since 23 December 2020 | | / |  |  |  | 0·104^***^  (0·062, 0·146) |
|  | Regular asymptomatic testing since 13 April 2021 | | / |  |  | -0·029  (-0·082, 0·023) | 0·029  (-0·013, 0·070) |
|  | Thrice-weekly LFD testing in staff since 15 December 2021 | | / |  |  |  | 0·014  (-0·042, 0·071) |
|  | Daily LFD testing in staff since 16 February 2022 | | / |  |  |  | 0·251^***^  (0·205, 0·296) |
| Testing intensity | Resident PCR | | (lag=1) | 0·043^***^  (0·041, 0·045) | 0·008^***^  (0·007, 0·009) | 0·568^***^  (0·566, 0·570) | 0·144^***^  (0·142, 0·147) |
|  |  | | (lag=2) | 0·053^***^  (0·051, 0·055) |  | 0·219^***^  (0·217, 0·221) |  |
|  |  | | (lag=1) | 0·041^***^  (0·039, 0·043) |  | 0·103^***^  (0·101, 0·106) |  |
|  |  | | (lag=2) | 0·283^***^  (0·281, 0·285) |  | 0·062^***^  (0·060, 0·064) |  |
|  |  | | (lag=5) | 0·092^***^  (0·090, 0·094) |  | 0·035^***^  (0·033, 0·037) |  |
|  | Resident LFD | | (lag=1) | 0·046^***^  (0·043, 0.048) | 0·442^***^  (0·440, 0·444) | -0·038^***^  (-0·039, -0·037) | 0·538^***^  (0·536, 0·540) |
|  |  | | (lag=2) |  | 0·119^***^  (0·117, 0·122) |  | 0·117^***^  (0·115, 0·120) |
|  |  | | (lag=3) |  | 0·047^***^  (0·044, 0·049) |  | 0·099^***^  (0·097, 0·102) |
|  |  | | (lag=4) |  | 0·067^***^  (0·065, 0·069) |  | -0·036^***^  (-0·038, -0·034) |
|  |  | | (lag=5) |  | 0·075^***^  (0·073, 0·077) |  | 0·162^***^  (0·160, 0·164) |
|  | Staff PCR | | (lag=1) | 0·042^***^  (0·042, 0·043) | -0·003^***^  (-0·003, -0·003) | 0·093^***^  (0·087, 0·098) | 0·021^***^  (0·014, 0·029) |
|  | Staff LFD | | (lag=1) | -0·008^***^  (-0·009, -0·008) | 0·003^***^  (0·003, 0·003) | -0·006^*^  (-0·013, 0·0004) | 0·057^***^  (0·047, 0·066) |

Note:^1^ *p<0·1; **p<0·05; ***p<0·01; p without * means p>0.1;

|  | PCR | 2,588,355,700 | 11% |
| --- | --- | --- | --- |
|  | Total | 3,797,232,900 | 16.2% |

# Table S5. Naïve regressions of numbers of positives in residents on lagged resident and staff tests.

Both regressions were Poisson models using log-links with no offset terms; coefficients and 95% confidence intervals are on the exponentiated scale.

|  | **Resident PCR + LFD positives count** | **Staff PCR + LFD positives count** |
| --- | --- | --- |
|  | **(1)** | **(2)** |
|  | | |
| Test intensity in residents (lag=1) | 1.507^***^ | 1.405^***^ |
|  | (1.507, 1.508) | (1.405, 1.405) |
|  |  |  |
| Test intensity in staff (lag=1) | 0.867^***^ | 1.035^***^ |
|  | (0.867, 0.867) | (1.035, 1.035) |
|  |  |  |
| Constant | 0.007^*^ | 0.000 |
|  | (-0.0002, 0.013) | (-0.00000, 0.00000) |
|  |  |  |
|  | | |
| Observations | 1,093,275 | 1,093,275 |
|  |  |  |
| *Note:* | *p<0.1; **p<0.05; ***p<0.01 | |

# Table S6. Outbreak discovery in residents – sensitivity analyses.

The dependent variable was the (non-zero) number of positives in residents in a given week if the previous week had zero positives. These results correspond to generalised linear models using a Poisson likelihood and a log-link function; coefficients and 95% confidence intervals are shown on the exponentiated scale. Estimates of the weekly time dummies and the effects associated with local COVID-19 prevalence are suppressed for readability.

|  | **Resident LFD + PCR positives count** | | | |
| --- | --- | --- | --- | --- |
|  | **1** | **2** | **3** | **4** |
|  | **(1)** | **(2)** | **(3)** | **(4)** |
|  | | | | |
| log(0.1 + test intensity in residents (lag=1)) | 0.943^***^ | 0.926^***^ |  |  |
|  | (0.933, 0.953) | (0.916, 0.936) |  |  |
|  |  |  |  |  |
| log(0.1 + test intensity in staff (lag=1)) | 0.775^***^ | 0.745^***^ |  |  |
|  | (0.766, 0.784) | (0.736, 0.755) |  |  |
|  |  |  |  |  |
| CQC: requires improvement |  | 0.990 |  | 0.999 |
|  |  | (0.925, 1.056) |  | (0.934, 1.065) |
|  |  |  |  |  |
| CQC: good |  | 0.904^***^ |  | 0.918^***^ |
|  |  | (0.840, 0.967) |  | (0.855, 0.982) |
|  |  |  |  |  |
| CQC: outstanding |  | 0.784^***^ |  | 0.812^***^ |
|  |  | (0.707, 0.861) |  | (0.735, 0.889) |
|  |  |  |  |  |
| CQC: null |  | 0.876^*^ |  | 0.857^**^ |
|  |  | (0.732, 1.020) |  | (0.713, 1.001) |
|  |  |  |  |  |
| Is nursing home? |  | 0.915^***^ |  | 0.910^***^ |
|  |  | (0.894, 0.936) |  | (0.889, 0.931) |
|  |  |  |  |  |
| Is independent? |  | 1.036 |  | 1.074 |
|  |  | (0.607, 1.465) |  | (0.645, 1.504) |
|  |  |  |  |  |
| Primary clients: older (≥65) individuals |  | 1.196^***^ |  | 1.181^***^ |
|  |  | (1.159, 1.233) |  | (1.144, 1.218) |
|  |  |  |  |  |
| Primary clients: individuals with dementia |  | 1.251^***^ |  | 1.234^***^ |
|  |  | (1.210, 1.292) |  | (1.193, 1.275) |
|  |  |  |  |  |
| Primary clients: individuals with learning disabilities |  | 1.246^***^ |  | 1.278^***^ |
|  |  | (1.202, 1.290) |  | (1.234, 1.322) |
|  |  |  |  |  |
| Local proportion of Delta variant |  | 1.285 |  | 1.293 |
|  |  | (0.698, 1.872) |  | (0.705, 1.882) |
|  |  |  |  |  |
| Local proportion of Omicron variant |  | 1.350^***^ |  | 1.376^***^ |
|  |  | (1.197, 1.504) |  | (1.222, 1.529) |
|  |  |  |  |  |
| log(staff per resident) |  | 1.037^***^ |  | 1.034^***^ |
|  |  | (1.012, 1.063) |  | (1.009, 1.060) |
|  |  |  |  |  |
| Fraction of agency workers |  | 1.077^***^ |  | 1.078^***^ |
|  |  | (1.042, 1.112) |  | (1.043, 1.114) |
|  |  |  |  |  |
| Is community care home? |  | 0.983 |  | 0.983 |
|  |  | (0.930, 1.036) |  | (0.930, 1.036) |
|  |  |  |  |  |
| Is acute care home? |  | 2.233^**^ |  | 2.137^**^ |
|  |  | (1.491, 2.975) |  | (1.395, 2.879) |
|  |  |  |  |  |
| Av. doses of all vaccines per resident |  | 0.990^***^ |  | 0.993^***^ |
|  |  | (0.986, 0.994) |  | (0.989, 0.996) |
|  |  |  |  |  |
| Av. doses of all vaccines per staff |  | 0.993^***^ |  | 0.994^***^ |
|  |  | (0.990, 0.996) |  | (0.991, 0.997) |
|  |  |  |  |  |
| Total resident count |  | 0.981^***^ |  | 0.981^***^ |
|  |  | (0.980, 0.982) |  | (0.980, 0.982) |
|  |  |  |  |  |
| log(0.1 + PCR test intensity in residents (lag=1)) |  |  | 0.987^**^ | 0.991^*^ |
|  |  |  | (0.976, 0.997) | (0.980, 1.001) |
|  |  |  |  |  |
| log(0.1 + LFD test intensity in residents (lag=1)) |  |  | 0.879^***^ | 0.820^***^ |
|  |  |  | (0.862, 0.897) | (0.802, 0.837) |
|  |  |  |  |  |
| log(0.1 + PCR test intensity in staff (lag=1)) |  |  | 0.819^***^ | 0.808^***^ |
|  |  |  | (0.807, 0.830) | (0.797, 0.819) |
|  |  |  |  |  |
| log(0.1 + LFD test intensity in staff (lag=1)) |  |  | 0.861^***^ | 0.833^***^ |
|  |  |  | (0.852, 0.870) | (0.824, 0.842) |
|  |  |  |  |  |
| Constant | 3.012^***^ | 7.922^***^ | 1.776^***^ | 3.913^***^ |
|  | (2.888, 3.136) | (7.777, 8.066) | (1.646, 1.906) | (3.763, 4.062) |
|  |  |  |  |  |
|  | | | | |
| Observations | 65,389 | 64,642 | 65,389 | 64,642 |
|  |  |  |  |  |
| *Note:* | *p<0.1; **p<0.05; ***p<0.01 | | | |

# Table S7. Outbreak discovery in staff – sensitivity analyses.

The dependent variable was the (non-zero) number of positives in staff in a given week if the previous week had zero positives. These results correspond to generalised linear models using a Poisson likelihood and a log-link function; coefficients and 95% confidence intervals are shown on the exponentiated scale. Estimates of the weekly time dummies and the effects associated with local COVID-19 prevalence are suppressed for readability.

|  | **Staff LFD + PCR positives count** | | | |
| --- | --- | --- | --- | --- |
|  | **1** | **2** | **3** | **4** |
|  | **(1)** | **(2)** | **(3)** | **(4)** |
|  | | | | |
| log(0.1 + test intensity in residents (lag=1)) | 0.987^***^ | 0.999 |  |  |
|  | (0.982, 0.993) | (0.994, 1.005) |  |  |
|  |  |  |  |  |
| log(0.1 + test intensity in staff (lag=1)) | 0.735^***^ | 0.662^***^ |  |  |
|  | (0.729, 0.742) | (0.655, 0.668) |  |  |
|  |  |  |  |  |
| CQC: requires improvement |  | 0.998 |  | 1.000 |
|  |  | (0.952, 1.044) |  | (0.954, 1.045) |
|  |  |  |  |  |
| CQC: good |  | 0.948^**^ |  | 0.954^**^ |
|  |  | (0.904, 0.993) |  | (0.909, 0.998) |
|  |  |  |  |  |
| CQC: outstanding |  | 0.857^***^ |  | 0.875^***^ |
|  |  | (0.807, 0.907) |  | (0.825, 0.925) |
|  |  |  |  |  |
| CQC: null |  | 0.935 |  | 0.891^**^ |
|  |  | (0.843, 1.028) |  | (0.799, 0.984) |
|  |  |  |  |  |
| Is nursing home? |  | 0.939^***^ |  | 0.928^***^ |
|  |  | (0.926, 0.952) |  | (0.915, 0.941) |
|  |  |  |  |  |
| Is independent? |  | 1.070 |  | 1.072 |
|  |  | (0.889, 1.251) |  | (0.891, 1.253) |
|  |  |  |  |  |
| Primary clients: older (≥65) individuals |  | 0.888^***^ |  | 0.882^***^ |
|  |  | (0.868, 0.909) |  | (0.861, 0.902) |
|  |  |  |  |  |
| Primary clients: individuals with dementia |  | 0.929^***^ |  | 0.916^***^ |
|  |  | (0.905, 0.952) |  | (0.893, 0.940) |
|  |  |  |  |  |
| Primary clients: individuals with learning disabilities |  | 1.199^***^ |  | 1.204^***^ |
|  |  | (1.176, 1.222) |  | (1.181, 1.227) |
|  |  |  |  |  |
| Local proportion of Delta variant |  | 0.999 |  | 1.016 |
|  |  | (0.743, 1.256) |  | (0.759, 1.273) |
|  |  |  |  |  |
| Local proportion of Omicron variant |  | 1.319^***^ |  | 1.298^***^ |
|  |  | (1.223, 1.414) |  | (1.202, 1.393) |
|  |  |  |  |  |
| log(staff per resident) |  | 1.031^***^ |  | 1.050^***^ |
|  |  | (1.017, 1.045) |  | (1.036, 1.064) |
|  |  |  |  |  |
| Fraction of agency workers |  | 1.071^***^ |  | 1.054^***^ |
|  |  | (1.040, 1.101) |  | (1.024, 1.085) |
|  |  |  |  |  |
| Is community care home? |  | 0.937^***^ |  | 0.930^***^ |
|  |  | (0.909, 0.966) |  | (0.902, 0.959) |
|  |  |  |  |  |
| Is acute care home? |  | 1.308 |  | 1.135 |
|  |  | (0.858, 1.759) |  | (0.684, 1.585) |
|  |  |  |  |  |
| Av. doses of all vaccines per resident |  | 1.001^***^ |  | 1.001^***^ |
|  |  | (1.000, 1.001) |  | (1.001, 1.001) |
|  |  |  |  |  |
| Av. doses of all vaccines per staff |  | 0.988^***^ |  | 0.991^***^ |
|  |  | (0.986, 0.990) |  | (0.989, 0.993) |
|  |  |  |  |  |
| Total resident count |  | 0.980^***^ |  | 0.980^***^ |
|  |  | (0.980, 0.981) |  | (0.980, 0.981) |
|  |  |  |  |  |
| log(0.1 + PCR test intensity in residents (lag=1)) |  |  | 0.971^***^ | 1.002 |
|  |  |  | (0.966, 0.977) | (0.996, 1.007) |
|  |  |  |  |  |
| log(0.1 + LFD test intensity in residents (lag=1)) |  |  | 1.041^***^ | 0.974^***^ |
|  |  |  | (1.030, 1.052) | (0.964, 0.985) |
|  |  |  |  |  |
| log(0.1 + PCR test intensity in staff (lag=1)) |  |  | 0.951^***^ | 0.878^***^ |
|  |  |  | (0.944, 0.959) | (0.871, 0.885) |
|  |  |  |  |  |
| log(0.1 + LFD test intensity in staff (lag=1)) |  |  | 0.744^***^ | 0.710^***^ |
|  |  |  | (0.739, 0.750) | (0.704, 0.716) |
|  |  |  |  |  |
| Constant | 3.950^***^ | 13.611^***^ | 2.258^***^ | 6.404^***^ |
|  | (3.882, 4.018) | (13.527, 13.695) | (2.186, 2.331) | (6.317, 6.492) |
|  |  |  |  |  |
|  | | | | |
| Observations | 94,558 | 93,485 | 94,558 | 93,485 |
| *Note:* | *p<0.1; **p<0.05; ***p<0.01 | | | |

# Table S8. Analysis of deviance for the resident outbreak discovery model.

This yields the reduction in residual deviance gained by adding each variable in turn (starting from the top) to the regression, so large values of “deviance” indicate a larger gain in predictive power.

| **Variable** | **Degrees of freedom (df)** | **Deviance** | **Residual df** | **Residual deviance** | **Significance level** |
| --- | --- | --- | --- | --- | --- |
| NULL | NA | NA | 64641 | 154508.2 | NA |
| log(0.1 + test intensity in residents (lag=1)) | 1 | 2596.9 | 64640 | 151911.3 | *** |
| log(0.1 + test intensity in staff (lag=1)) | 1 | 5401.5 | 64639 | 146509.7 | *** |
| CQC: requires improvement | 1 | 23.2 | 64638 | 146486.6 | *** |
| CQC: good | 1 | 25.5 | 64637 | 146461.0 | *** |
| CQC: outstanding | 1 | 96.5 | 64636 | 146364.5 | *** |
| CQC: null | 1 | 9.9 | 64635 | 146354.6 | *** |
| Is nursing home? | 1 | 1668.5 | 64634 | 144686.1 | *** |
| Is independent? | 1 | 0.1 | 64633 | 144686.0 |  |
| Primary clients: older (≥65) individuals | 1 | 100.9 | 64632 | 144585.1 | *** |
| Primary clients: individuals with dementia | 1 | 396.4 | 64631 | 144188.7 | *** |
| Primary clients: individuals with learning disabilities | 1 | 295.1 | 64630 | 143893.6 | *** |
| Local proportion of Delta variant | 1 | 40.8 | 64629 | 143852.8 | *** |
| Local proportion of Omicron variant | 1 | 1684.7 | 64628 | 142168.1 | *** |
| log(staff per resident) | 1 | 575.0 | 64627 | 141593.1 | *** |
| Fraction of agency workers | 1 | 41.5 | 64626 | 141551.6 | *** |
| Local COVID-19 prevalence | 1 | 6487.0 | 64625 | 135064.6 | *** |
| Is community care home? | 1 | 10.3 | 64624 | 135054.2 | *** |
| Is acute care home? | 1 | 4.3 | 64623 | 135050.0 | ** |
| Av. doses of all vaccines per resident | 1 | 1157.1 | 64622 | 133892.9 | *** |
| Av. doses of all vaccines per staff | 1 | 483.9 | 64621 | 133409.0 | *** |
| Total resident count | 1 | 4276.6 | 64620 | 129132.4 | *** |
| Week time dummies | 76 | 5799.8 | 64544 | 123332.6 | *** |

# Table S9. Analysis of deviance for the staff outbreak discovery model.

This yields the reduction in residual deviance gained by adding each variable in turn (starting from the top) to the regression, so large values of “deviance” indicate a larger gain in predictive power.

| **Variable** | **Degrees of freedom (df)** | **Deviance** | **Residual df** | **Residual deviance** | **Significance level** |
| --- | --- | --- | --- | --- | --- |
| NULL | NA | NA | 93484 | 186843.1 | NA |
| log(0.1 + test intensity in residents (lag=1)) | 1 | 1964.3 | 93483 | 184878.8 | *** |
| log(0.1 + test intensity in staff (lag=1)) | 1 | 16871.1 | 93482 | 168007.7 | *** |
| CQC: requires improvement | 1 | 0.1 | 93481 | 168007.6 |  |
| CQC: good | 1 | 112.2 | 93480 | 167895.4 | *** |
| CQC: outstanding | 1 | 127.0 | 93479 | 167768.4 | *** |
| CQC: null | 1 | 16.6 | 93478 | 167751.7 | *** |
| Is nursing home? | 1 | 5985.7 | 93477 | 161766.1 | *** |
| Is independent? | 1 | 14.2 | 93476 | 161751.9 | *** |
| Primary clients: older (≥65) individuals | 1 | 2719.3 | 93475 | 159032.6 | *** |
| Primary clients: individuals with dementia | 1 | 4442.0 | 93474 | 154590.6 | *** |
| Primary clients: individuals with learning disabilities | 1 | 945.0 | 93473 | 153645.7 | *** |
| Local proportion of Delta variant | 1 | 873.2 | 93472 | 152772.5 | *** |
| Local proportion of Omicron variant | 1 | 10161.4 | 93471 | 142611.1 | *** |
| log(staff per resident) | 1 | 1249.6 | 93470 | 141361.5 | *** |
| Fraction of agency workers | 1 | 37.6 | 93469 | 141323.9 | *** |
| Local COVID-19 prevalence | 1 | 10006.1 | 93468 | 131317.8 | *** |
| Is community care home? | 1 | 82.8 | 93467 | 131235.0 | *** |
| Is acute care home? | 1 | 4.7 | 93466 | 131230.3 | ** |
| Av. doses of all vaccines per resident | 1 | 28.6 | 93465 | 131201.7 | *** |
| Av. doses of all vaccines per staff | 1 | 842.7 | 93464 | 130358.9 | *** |
| Total resident count | 1 | 10459.3 | 93463 | 119899.6 | *** |
| Week time dummies | 76 | 16948.2 | 93387 | 102951.4 | *** |

# Table S10. Outbreak discovery in residents – date sensitivity analyses.

The dependent variable was the (non-zero) number of positives in residents in a given week if the previous week had zero positives. Each regression corresponds to data from a distinct time-period, as indicated at the top of the columns. These results correspond to generalised linear models using a Poisson likelihood and a log-link function; coefficients and 95% confidence intervals are shown on the exponentiated scale. Estimates of the weekly time dummies are suppressed for readability.

|  | **Resident LFD + PCR positives count** | | |
| --- | --- | --- | --- |
|  | **1 October 2020 to 31 December 2020** | **1 January 2021 to 30 November 2021** | **1 December 2021 to 31 March 2022** |
|  | **(1)** | **(2)** | **(3)** |
|  | | | |
| log(0.1 + test intensity in residents (lag=1)) | 1.063^***^ | 0.992 | 0.861^***^ |
|  | (1.032, 1.095) | (0.976, 1.007) | (0.847, 0.875) |
|  |  |  |  |
| log(0.1 + test intensity in staff (lag=1)) | 0.746^***^ | 0.734^***^ | 0.747^***^ |
|  | (0.725, 0.768) | (0.717, 0.750) | (0.733, 0.761) |
|  |  |  |  |
| CQC: requires improvement | 1.158^*^ | 0.792^***^ | 1.185^***^ |
|  | (0.990, 1.326) | (0.700, 0.885) | (1.071, 1.298) |
|  |  |  |  |
| CQC: good | 1.031 | 0.717^***^ | 1.102^*^ |
|  | (0.868, 1.193) | (0.629, 0.806) | (0.991, 1.213) |
|  |  |  |  |
| CQC: outstanding | 0.875 | 0.575^***^ | 1.014 |
|  | (0.673, 1.077) | (0.462, 0.688) | (0.887, 1.141) |
|  |  |  |  |
| CQC: null | 0.915 | 0.838^*^ | 0.890 |
|  | (0.447, 1.383) | (0.639, 1.037) | (0.653, 1.128) |
|  |  |  |  |
| Is nursing home? | 0.848^***^ | 1.011 | 0.850^***^ |
|  | (0.795, 0.901) | (0.978, 1.044) | (0.818, 0.881) |
|  |  |  |  |
| Is independent? | 0.00002 | 0.645 | 1.535 |
|  | (-192.781, 192.781) | (-0.011, 1.300) | (0.963, 2.107) |
|  |  |  |  |
| Primary clients: older (≥65) individuals | 1.285^***^ | 1.324^***^ | 1.015 |
|  | (1.184, 1.386) | (1.265, 1.384) | (0.962, 1.068) |
|  |  |  |  |
| Primary clients: individuals with dementia | 1.546^***^ | 1.356^***^ | 1.046 |
|  | (1.436, 1.656) | (1.290, 1.422) | (0.986, 1.105) |
|  |  |  |  |
| Primary clients: individuals with learning disabilities | 1.212^***^ | 1.229^***^ | 1.295^***^ |
|  | (1.088, 1.336) | (1.157, 1.301) | (1.233, 1.358) |
|  |  |  |  |
| Local proportion of Delta variant |  | 1.525 | 0.00001^***^ |
|  |  | (0.942, 2.108) | (-3.516, 3.516) |
|  |  |  |  |
| Local proportion of Omicron variant |  | 0.000 | 1.095 |
|  |  | (-75.175, 75.175) | (0.941, 1.248) |
|  |  |  |  |
| log(staff per resident) | 0.979 | 0.906^***^ | 1.210^***^ |
|  | (0.920, 1.038) | (0.866, 0.947) | (1.171, 1.250) |
|  |  |  |  |
| Fraction of agency workers | 0.772^***^ | 1.062^***^ | 1.137^***^ |
|  | (0.575, 0.968) | (1.018, 1.107) | (1.071, 1.202) |
|  |  |  |  |
| Local COVID-19 prevalence | 0.000^***^ | 0.000^***^ | 0.00003^***^ |
|  | (0.000,0.000) | (0.000,0.000) | (0.000,0.000) |
|  |  |  |  |
| Is community care home? | 1.096 | 0.930 | 0.966 |
|  | (0.976, 1.217) | (0.843, 1.018) | (0.885, 1.047) |
|  |  |  |  |
| Is acute care home? |  | 2.173 | 3.327^***^ |
|  |  | (0.786, 3.560) | (2.446, 4.207) |
|  |  |  |  |
| Av. doses of all vaccines per resident | 0.779^**^ | 0.999 | 0.993^***^ |
|  | (0.554, 1.005) | (0.994, 1.005) | (0.989, 0.998) |
|  |  |  |  |
| Av. doses of all vaccines per staff | 0.952 | 0.988^***^ | 0.991^***^ |
|  | (0.862, 1.042) | (0.981, 0.996) | (0.987, 0.994) |
|  |  |  |  |
| Total resident count | 0.981^***^ | 0.978^***^ | 0.985^***^ |
|  | (0.979, 0.982) | (0.977, 0.979) | (0.984, 0.986) |
|  |  |  |  |
| Constant | 11.500^***^ | 55.644^***^ | 3.325^***^ |
|  | (11.262, 11.739) | (55.511, 55.777) | (3.131, 3.520) |
|  |  |  |  |
|  | | | |
| Observations | 8,183 | 32,915 | 23,544 |
| *Note:* | *p<0.1; **p<0.05; ***p<0.01 | | |

# Table S11. Outbreak discovery in staff – date sensitivity analyses.

The dependent variable was the (non-zero) number of positives in staff in a given week if the previous week had zero positives. Each regression corresponds to data from a distinct time-period, as indicated at the top of the columns. These results correspond to generalised linear models using a Poisson likelihood and a log-link function; coefficients and 95% confidence intervals are shown on the exponentiated scale. Estimates of the weekly time dummies are suppressed for readability.

|  | **Staff LFD + PCR positives count** | | |
| --- | --- | --- | --- |
|  | **1 October 2020 to 31 December 2020** | **1 January 2021 to 30 November 2021** | **1 December 2021 to 31 March 2022** |
|  | **(1)** | **(2)** | **(3)** |
|  | | | |
| log(0.1 + test intensity in residents (lag=1)) | 0.989 | 1.014^***^ | 0.998 |
|  | (0.971, 1.007) | (1.005, 1.022) | (0.990, 1.006) |
|  |  |  |  |
| log(0.1 + test intensity in staff (lag=1)) | 0.836^***^ | 0.661^***^ | 0.582^***^ |
|  | (0.821, 0.851) | (0.651, 0.672) | (0.573, 0.592) |
|  |  |  |  |
| CQC: requires improvement | 0.979 | 0.933^**^ | 1.036 |
|  | (0.853, 1.106) | (0.863, 1.002) | (0.966, 1.107) |
|  |  |  |  |
| CQC: good | 0.963 | 0.882^***^ | 0.990 |
|  | (0.841, 1.085) | (0.815, 0.950) | (0.922, 1.058) |
|  |  |  |  |
| CQC: outstanding | 0.923 | 0.782^***^ | 0.920^**^ |
|  | (0.783, 1.062) | (0.707, 0.857) | (0.843, 0.996) |
|  |  |  |  |
| CQC: null | 0.650^**^ | 0.880^*^ | 1.002 |
|  | (0.265, 1.036) | (0.740, 1.019) | (0.869, 1.136) |
|  |  |  |  |
| Is nursing home? | 0.870^***^ | 0.939^***^ | 0.945^***^ |
|  | (0.833, 0.907) | (0.920, 0.959) | (0.926, 0.965) |
|  |  |  |  |
| Is independent? | 1.169 | 1.021 | 1.153 |
|  | (0.689, 1.650) | (0.775, 1.267) | (0.830, 1.477) |
|  |  |  |  |
| Primary clients: older (≥65) individuals | 0.904^***^ | 0.911^***^ | 0.854^***^ |
|  | (0.843, 0.965) | (0.879, 0.942) | (0.824, 0.884) |
|  |  |  |  |
| Primary clients: individuals with dementia | 0.997 | 0.943^***^ | 0.883^***^ |
|  | (0.928, 1.066) | (0.907, 0.978) | (0.847, 0.918) |
|  |  |  |  |
| Primary clients: individuals with learning disabilities | 1.251^***^ | 1.222^***^ | 1.151^***^ |
|  | (1.181, 1.320) | (1.186, 1.257) | (1.117, 1.185) |
|  |  |  |  |
| Local proportion of Delta variant |  | 0.940 | 0.015^***^ |
|  |  | (0.681, 1.199) | (-1.570, 1.599) |
|  |  |  |  |
| Local proportion of Omicron variant |  | 0.000 | 1.122^**^ |
|  |  | (-36.937, 36.937) | (1.026, 1.217) |
|  |  |  |  |
| log(staff per resident) | 1.301^***^ | 1.060^***^ | 0.883^***^ |
|  | (1.262, 1.340) | (1.039, 1.082) | (0.861, 0.904) |
|  |  |  |  |
| Fraction of agency workers | 0.786^***^ | 1.096^***^ | 1.058^**^ |
|  | (0.661, 0.910) | (1.056, 1.136) | (1.013, 1.102) |
|  |  |  |  |
| Local COVID-19 prevalence | 0.000^***^ | 0.000^***^ | 0.000^***^ |
|  | (0.000,0.000) | (0.000,0.000) | (0.000,0.000) |
|  |  |  |  |
| Is community care home? | 0.960 | 0.913^***^ | 0.967 |
|  | (0.883, 1.038) | (0.869, 0.957) | (0.923, 1.010) |
|  |  |  |  |
| Is acute care home? | 0.637 | 1.577 | 1.722 |
|  | (-0.241, 1.515) | (0.775, 2.378) | (1.028, 2.416) |
|  |  |  |  |
| Av. doses of all vaccines per resident | 0.941 | 0.999 | 1.001^***^ |
|  | (0.844, 1.039) | (0.996, 1.002) | (1.000, 1.001) |
|  |  |  |  |
| Av. doses of all vaccines per staff | 0.980 | 0.977^***^ | 0.999 |
|  | (0.917, 1.044) | (0.973, 0.981) | (0.997, 1.001) |
|  |  |  |  |
| Total resident count | 0.978^***^ | 0.978^***^ | 0.984^***^ |
|  | (0.977, 0.979) | (0.978, 0.979) | (0.983, 0.984) |
|  |  |  |  |
| Constant | 24.648^***^ | 34.970^***^ | 5.451^***^ |
|  | (24.489, 24.807) | (34.879, 35.061) | (5.349, 5.554) |
|  |  |  |  |
|  | | | |
| Observations | 12,033 | 47,323 | 34,129 |
| *Note:* | *p<0.1; **p<0.05; ***p<0.01 | | |

# Table S12. Regression results for determinants of weekly outbreak size in residents and staff (measured by number of positives) during outbreaks: sensitivity analysis.

These results correspond to generalised linear models using a Poisson likelihood and a log-link function; coefficients and 95% confidence intervals are shown on the exponentiated scale. Estimates of the weekly time dummies and effect size associated with local COVID-19 prevalence are suppressed for readability.

|  | **Resident PCR + LFD positives count** | **Staff PCR + LFD positives count** |
| --- | --- | --- |
|  | **(1)** | **(2)** |
|  | | |
| Num. positives/care home member (lag=1) | 0.274^***^ | 0.193^***^ |
|  | (0.236, 0.313) | (0.159, 0.227) |
|  |  |  |
| Fraction of positives in residents (lag=1) | 1.676^***^ | 0.782^***^ |
|  | (1.656, 1.695) | (0.766, 0.799) |
|  |  |  |
| log(0.1 + test intensity in residents (lag=1)) | 0.719^***^ | 0.933^***^ |
|  | (0.712, 0.725) | (0.929, 0.938) |
|  |  |  |
| log(0.1 + test intensity in staff (lag=1)) | 0.767^***^ | 0.528^***^ |
|  | (0.759, 0.776) | (0.521, 0.535) |
|  |  |  |
| CQC: requires improvement | 0.937^***^ | 0.911^***^ |
|  | (0.896, 0.979) | (0.875, 0.947) |
|  |  |  |
| CQC: good | 0.916^***^ | 0.903^***^ |
|  | (0.876, 0.956) | (0.868, 0.938) |
|  |  |  |
| CQC: outstanding | 0.850^***^ | 0.879^***^ |
|  | (0.804, 0.896) | (0.841, 0.917) |
|  |  |  |
| CQC: null | 0.880^***^ | 0.925^**^ |
|  | (0.791, 0.969) | (0.859, 0.991) |
|  |  |  |
| Is nursing home? | 0.921^***^ | 1.018^***^ |
|  | (0.909, 0.933) | (1.009, 1.028) |
|  |  |  |
| Is independent? | 1.111 | 1.072 |
|  | (0.917, 1.304) | (0.980, 1.163) |
|  |  |  |
| Primary clients: older (≥65) individuals | 1.316^***^ | 1.039^***^ |
|  | (1.292, 1.339) | (1.023, 1.055) |
|  |  |  |
| Primary clients: individuals with dementia | 1.305^***^ | 1.058^***^ |
|  | (1.280, 1.330) | (1.041, 1.076) |
|  |  |  |
| Primary clients: individuals with learning disabilities | 0.991 | 0.948^***^ |
|  | (0.961, 1.022) | (0.928, 0.968) |
|  |  |  |
| Local proportion of Delta variant | 0.519^**^ | 0.449^***^ |
|  | (-0.105, 1.142) | (-0.024, 0.922) |
|  |  |  |
| Local proportion of Omicron variant | 0.931^*^ | 1.072^**^ |
|  | (0.859, 1.003) | (1.018, 1.126) |
|  |  |  |
| log(staff per resident) | 0.753^***^ | 0.617^***^ |
|  | (0.737, 0.770) | (0.605, 0.629) |
|  |  |  |
| Fraction of agency workers | 1.028^*^ | 0.987 |
|  | (1.000, 1.055) | (0.962, 1.012) |
|  |  |  |
| Is community care home? | 1.016 | 1.024^**^ |
|  | (0.987, 1.046) | (1.004, 1.044) |
|  |  |  |
| Is acute care home? | 0.974 | 0.766 |
|  | (0.430, 1.518) | (0.316, 1.216) |
|  |  |  |
| Av. doses of all vaccines per resident | 0.983^***^ | 0.997^***^ |
|  | (0.980, 0.985) | (0.996, 0.999) |
|  |  |  |
| Av. doses of all vaccines per staff | 0.995^***^ | 1.001 |
|  | (0.993, 0.996) | (1.000, 1.002) |
|  |  |  |
| Total resident count | 1.001^***^ | 1.003^***^ |
|  | (1.001, 1.001) | (1.002, 1.003) |
|  |  |  |
| Constant | 0.488^***^ | 0.531^***^ |
|  | (0.338, 0.639) | (0.409, 0.653) |
|  |  |  |
|  | | |
| Observations | 117,988 | 164,546 |
| *Note:* | *p<0.1; **p<0.05; ***p<0.01 | |

# Table S13. Regression results for determinants of weekly outbreak size in residents (measured by number of positives) during outbreaks: time-period sensitivity analysis.

These results correspond to generalised linear models using a Poisson likelihood and a log-link function; coefficients and 95% confidence intervals are shown on the exponentiated scale. Estimates of the weekly time dummies are suppressed for readability. Note that the Omicron variant variable and local COVID-19 prevalence have been dropped from these regressions as the coefficients associated with these variables were unrealistically large, likely reflecting the small sample sizes and lack of variation among these variables within certain periods.

|  | **1 October 2020 to 31 December 2020** | **1 January 2021 to 30 November 2021** | **1 December 2021 to 31 March 2022** |
| --- | --- | --- | --- |
|  | **(1)** | **(2)** | **(3)** |
|  | | | |
| Fraction of positives in residents (lag=1) | 1.278^***^ | 1.521^***^ | 1.500^***^ |
|  | (1.212, 1.344) | (1.485, 1.557) | (1.473, 1.527) |
|  |  |  |  |
| log(0.1 + test intensity in residents (lag=1)) | 0.729^***^ | 0.702^***^ | 0.658^***^ |
|  | (0.707, 0.752) | (0.691, 0.714) | (0.650, 0.666) |
|  |  |  |  |
| log(0.1 + test intensity in staff (lag=1)) | 0.719^***^ | 0.792^***^ | 0.700^***^ |
|  | (0.683, 0.756) | (0.777, 0.808) | (0.690, 0.711) |
|  |  |  |  |
| CQC: requires improvement | 1.328^***^ | 0.839^***^ | 1.003 |
|  | (1.162, 1.494) | (0.774, 0.904) | (0.946, 1.060) |
|  |  |  |  |
| CQC: good | 1.362^***^ | 0.807^***^ | 1.006 |
|  | (1.199, 1.525) | (0.744, 0.869) | (0.951, 1.061) |
|  |  |  |  |
| CQC: outstanding | 1.170^*^ | 0.775^***^ | 0.957 |
|  | (0.990, 1.350) | (0.700, 0.850) | (0.895, 1.020) |
|  |  |  |  |
| CQC: null | 1.306 | 1.057 | 0.899^*^ |
|  | (0.945, 1.668) | (0.886, 1.228) | (0.788, 1.010) |
|  |  |  |  |
| Is nursing home? | 0.904^***^ | 0.933^***^ | 0.937^***^ |
|  | (0.866, 0.942) | (0.911, 0.954) | (0.922, 0.952) |
|  |  |  |  |
| Is independent? | 0.818 | 1.541^***^ | 0.913 |
|  | (0.353, 1.283) | (1.246, 1.836) | (0.604, 1.221) |
|  |  |  |  |
| Primary clients: older (≥65) individuals | 1.607^***^ | 1.348^***^ | 1.219^***^ |
|  | (1.523, 1.692) | (1.306, 1.390) | (1.189, 1.249) |
|  |  |  |  |
| Primary clients: individuals with dementia | 1.475^***^ | 1.242^***^ | 1.259^***^ |
|  | (1.385, 1.565) | (1.196, 1.288) | (1.227, 1.291) |
|  |  |  |  |
| Primary clients: individuals with learning disabilities | 0.919 | 0.830^***^ | 1.039^*^ |
|  | (0.812, 1.026) | (0.773, 0.886) | (1.001, 1.078) |
|  |  |  |  |
| Local proportion of Delta variant |  | 0.635 | 0.001^***^ |
|  |  | (0.007, 1.262) | (-2.883, 2.885) |
|  |  |  |  |
| log(staff per resident) | 0.626^***^ | 0.603^***^ | 0.789^***^ |
|  | (0.575, 0.677) | (0.574, 0.632) | (0.767, 0.811) |
|  |  |  |  |
| Fraction of agency workers | 0.704^***^ | 1.014 | 1.031 |
|  | (0.524, 0.885) | (0.967, 1.061) | (0.993, 1.069) |
|  |  |  |  |
| Is community care home? | 0.840^***^ | 1.139^***^ | 1.005 |
|  | (0.744, 0.936) | (1.086, 1.192) | (0.966, 1.043) |
|  |  |  |  |
| Is acute care home? | 1.797 | 0.806 | 1.838^*^ |
|  | (-0.167, 3.761) | (-0.174, 1.787) | (1.144, 2.532) |
|  |  |  |  |
| Av. doses of all vaccines per resident | 1.007 | 0.979^***^ | 0.991^***^ |
|  | (0.938, 1.077) | (0.973, 0.985) | (0.988, 0.993) |
|  |  |  |  |
| Av. doses of all vaccines per staff | 1.030 | 0.988^***^ | 0.991^***^ |
|  | (0.993, 1.067) | (0.982, 0.995) | (0.990, 0.993) |
|  |  |  |  |
| Total resident count | 1.001^*^ | 1.003^***^ | 1.004^***^ |
|  | (1.000, 1.002) | (1.003, 1.004) | (1.003, 1.004) |
|  |  |  |  |
| Constant | 0.333^***^ | 1.363^***^ | 0.392^***^ |
|  | (0.097, 0.568) | (1.279, 1.447) | (0.243, 0.541) |
|  |  |  |  |
|  | | | |
| Observations | 11,111 | 51,026 | 55,851 |
| *Note:* | *p<0.1; **p<0.05; ***p<0.01 | | |

**Table S14. Regression results for determinants of weekly outbreak size in staff (measured by number of positives) during outbreaks: time-period sensitivity analysis.**

These results correspond to generalised linear models using a Poisson likelihood and a log-link function; coefficients and 95% confidence intervals are shown on the exponentiated scale. Estimates of the weekly time dummies are suppressed for readability. Note that the Omicron variant variable and local COVID-19 prevalence have been dropped from these regressions as the coefficients associated with these variables were unrealistically large, likely reflecting the small sample sizes and lack of variation among these variables within certain periods.

|  | **1 October 2020 to 31 December 2020** | **1 January 2021 to 30 November 2021** | **1 December 2021 to 31 March 2022** |
| --- | --- | --- | --- |
|  | **(1)** | **(2)** | **(3)** |
|  | | | |
| Fraction of positives in residents (lag=1) | 1.010 | 0.933^***^ | 0.466^***^ |
|  | (0.950, 1.070) | (0.902, 0.965) | (0.445, 0.487) |
|  |  |  |  |
| log(0.1 + test intensity in residents (lag=1)) | 0.816^***^ | 0.883^***^ | 0.910^***^ |
|  | (0.796, 0.835) | (0.873, 0.893) | (0.904, 0.915) |
|  |  |  |  |
| log(0.1 + test intensity in staff (lag=1)) | 0.673^***^ | 0.574^***^ | 0.445^***^ |
|  | (0.641, 0.706) | (0.560, 0.587) | (0.437, 0.454) |
|  |  |  |  |
| CQC: requires improvement | 1.147^*^ | 0.809^***^ | 0.971 |
|  | (1.001, 1.292) | (0.746, 0.872) | (0.925, 1.017) |
|  |  |  |  |
| CQC: good | 1.111 | 0.766^***^ | 0.988 |
|  | (0.970, 1.253) | (0.705, 0.827) | (0.943, 1.033) |
|  |  |  |  |
| CQC: outstanding | 1.028 | 0.758^***^ | 0.976 |
|  | (0.873, 1.183) | (0.690, 0.827) | (0.927, 1.024) |
|  |  |  |  |
| CQC: null | 0.795 | 0.761^***^ | 1.049 |
|  | (0.432, 1.158) | (0.599, 0.924) | (0.973, 1.125) |
|  |  |  |  |
| Is nursing home? | 1.014 | 0.994 | 1.053^***^ |
|  | (0.977, 1.050) | (0.975, 1.013) | (1.041, 1.064) |
|  |  |  |  |
| Is independent? | 1.144 | 1.082 | 0.972 |
|  | (0.809, 1.478) | (0.900, 1.264) | (0.861, 1.083) |
|  |  |  |  |
| Primary clients: older (≥65) individuals | 1.136^***^ | 1.090^***^ | 1.029^***^ |
|  | (1.067, 1.204) | (1.056, 1.125) | (1.009, 1.048) |
|  |  |  |  |
| Primary clients: individuals with dementia | 1.168^***^ | 1.077^***^ | 1.041^***^ |
|  | (1.094, 1.242) | (1.039, 1.114) | (1.020, 1.062) |
|  |  |  |  |
| Primary clients: individuals with learning disabilities | 0.894^***^ | 0.853^***^ | 0.941^***^ |
|  | (0.812, 0.975) | (0.811, 0.895) | (0.918, 0.965) |
|  |  |  |  |
| Local proportion of Delta variant |  | 0.585^**^ | 0.003^***^ |
|  |  | (0.109, 1.060) | (-1.959, 1.964) |
|  |  |  |  |
| log(staff per resident) | 0.679^***^ | 0.549^***^ | 0.567^***^ |
|  | (0.634, 0.723) | (0.526, 0.573) | (0.552, 0.581) |
|  |  |  |  |
| Fraction of agency workers | 0.687^***^ | 0.959 | 0.972^**^ |
|  | (0.540, 0.834) | (0.904, 1.013) | (0.943, 1.000) |
|  |  |  |  |
| Is community care home? | 0.959 | 1.052^**^ | 1.024^*^ |
|  | (0.884, 1.035) | (1.011, 1.092) | (1.000, 1.047) |
|  |  |  |  |
| Is acute care home? | 0.843 | 1.607 | 0.547 |
|  | (-0.139, 1.825) | (0.987, 2.228) | (-0.329, 1.424) |
|  |  |  |  |
| Av. doses of all vaccines per resident | 0.965 | 0.984^***^ | 1.000 |
|  | (0.902, 1.028) | (0.979, 0.990) | (0.999, 1.001) |
|  |  |  |  |
| Av. doses of all vaccines per staff | 0.981 | 1.005^***^ | 1.000 |
|  | (0.946, 1.017) | (1.001, 1.009) | (0.999, 1.001) |
|  |  |  |  |
| Total resident count | 1.004^***^ | 1.006^***^ | 1.004^***^ |
|  | (1.003, 1.004) | (1.006, 1.007) | (1.004, 1.005) |
|  |  |  |  |
| Constant | 0.291^***^ | 0.970 | 0.331^***^ |
|  | (0.094, 0.488) | (0.893, 1.047) | (0.215, 0.447) |
|  |  |  |  |
|  | | | |
| Observations | 15,778 | 72,467 | 76,301 |
| *Note:* | *p<0.1; **p<0.05; ***p<0.01 | | |

# Table S15. Summary of the costs and cost effectiveness of the testing programme in care homes compared with hypothetical changes in the testing volume for the financial years FY21 and FY22.

|  | **Full evaluation period (October 2020–March 2022)** | | | **FY21**  **(October 2020–March 2022)** | | | **FY22**  **(April 2021–March 2022)** | | |
| --- | --- | --- | --- | --- | --- | --- | --- | --- | --- |
|  | Baseline compared with percentage testing volume | | | Baseline compared with percentage testing volume | | | Baseline compared with percentage testing volume | | |
| Comparator | 50% | 150% | 200% | 50% | 150% | 200% | 50% | 150% | 200% |
| Cost (GBP billions)^1^ | 2·56 | 5·03 | 6·26 | 1·40 | 2·76 | 3·45 | 1·16 | 2·05 | 2·74 |
| Cost per death averted, in residents only, due to testing (GBP) | 38,300 | 154,100 | 210,200 | 28,300 | 103,000 | 142,500 | 68,000 | 396,600 | 507,200 |
| Cost per QALY gained from deaths averted in residents due to testing^​2^ | 5,700 | 22,700 | 31,000 | 4200 | 15,200 | 21,000 | 10,000 | 58,500 | 74,800 |
| ^1^Baseline (actual) costs: FY21 = GBP 2·09 billion; FY22 = GBP 1·71 billion; full evaluation period = GBP 3·80 billion  ^2^A QALY of 6·78 per death was used  FY, financial year; QALY, quality-adjusted life-year | | | | | | | | | |
|  | | | | | | | | | |

# Table S16. Data inputs and assumptions for the testing service in adult social care

| **Parameter** | **Value** | **Source** |
| --- | --- | --- |
| Hospitalisation fatality ratio (HFR) | 20.13 | Calculated from ONS data (deaths/hospitalisations)^33^ |
| QALYs for death | 6.78 (4.98–8.8) | Sandmann et al., 2021^34^; UKHSA confidential internal documents |
| QALYs for hospitalisations | 0.201 | Sandmann et al., 2021^34^; UKHSA confidential internal documents |
| QALYs for admission to an intensive care unit (ICU) | 0.15 | Sandmann et al., 2021^34^; UKHSA confidential internal documents |
| QALYs for symptomatic COVID-19 infections | 0.008 | DHSC, 2020^35^ |
| Proportion of hospitalised patients with major manifestations | 0.41 (≥19 years)  0.2 (≤18 years) | EY–Oxford Health Analytics  Consortium, 2023^36^ |
| Proportion of hospitalised patients with pneumonia | 0.42 (≥19 years)  0.11 (≤18 years) | EY–Oxford Health Analytics  Consortium, 2023^36^ |
| Proportion of hospitalised patients with major manifestations or pneumonia in ICU | 0.11 (≥19 years)  0.9 (≤18 years) | EY–Oxford Health Analytics  Consortium, 2023^36^ |
| Cost of hospitalisation (GBP) | 2771 (≥19 years)  3138 (≤18 years) | NHS Schedule of Costs^37^ |
| Cost of hospitalisation with major manifestations (GBP) | 4507 (≥19 years)  8606 (≤18 years) | NHS Schedule of Costs^37^ |

# Supplementary Materials – References

1. Public Health England. Guidance for social or community care and residential settings on COVID-19 [Withdrawn] 2020 [Available from: <https://www.gov.uk/government/publications/guidance-for-social-or-community-care-and-residential-settings-on-covid-19/guidance-for-social-or-community-care-and-residential-settings-on-covid-19> accessed 29 March 2024.

2. NHS England. COVID-19 Hospital Discharge Service Requirements (letter) 2020 [Available from: <https://www.england.nhs.uk/coronavirus/wp-content/uploads/sites/52/2020/03/hmg-letter-hospital-discharge-guidance-v3.pdf> accessed 29 March 2024.

3. NHS. COVID-19 Hospital Discharge Service Requirements 2020 [Available from: <https://assets.publishing.service.gov.uk/government/uploads/system/uploads/attachment_data/file/911541/COVID-19_hospital_discharge_service_requirements_2.pdf> accessed 29 March 2024.

4. Department of Health and Social Care, Care Quality Commission, UK Health Security Agency. Coronavirus (COVID-19): admission and care of people in care homes [Withdrawn] 2020 [Available from: <https://www.gov.uk/government/publications/coronavirus-covid-19-admission-and-care-of-people-in-care-homes> accessed 19 October 2022.

5. Department of Health and Social Care. COVID-19: our action plan for adult social care 2020 [Available from: <https://www.gov.uk/government/publications/coronavirus-covid-19-adult-social-care-action-plan/covid-19-our-action-plan-for-adult-social-care> accessed 19 October 2022.

6. Department of Health and social Care. Government to offer testing for “everyone who needs one” in social care settings 2020 [Available from: <https://www.gov.uk/government/news/government-to-offer-testing-for-everyone-who-needs-one-in-social-care-settings> accessed 29 March 2024.

7. NHS England. New requirement to test patients being discharged from hospital to a care home (letter) 2020 [Available from: <https://www.england.nhs.uk/coronavirus/wp-content/uploads/sites/52/2020/04/C0324-New-requirement-to-test-patients-being-discharged-from-hospital-to-a-care-home.pdf> accessed 29 March 2024.

8. Department of Health and Social Care. Coronavirus testing extended to all essential workers in England who have symptoms 2020 [Available from: <https://www.gov.uk/government/news/coronavirus-testing-extended-to-all-essential-workers-in-england-who-have-symptoms> accessed 29 March 2024.

9. Action for Carers Surrey. Unpaid carers given essential worker status 2020 [Available from: <https://www.actionforcarers.org.uk/news/unpaid-carers-given-essential-worker-status/> accessed 1 March 2023.

10. Department of Health and Social Care. Further expansion of access to coronavirus testing helps protect the most vulnerable 2020 [Available from: <https://www.gov.uk/government/news/further-expansion-of-access-to-coronavirus-testing-helps-protect-the-most-vulnerable> accessed 29 March 2024.

11. Department of Health and Social Care. Government launches new portal for care homes to arrange coronavirus testing 2020 [Available from: <https://www.gov.uk/government/news/government-launches-new-portal-for-care-homes-to-arrange-coronavirus-testing> accessed 29 March 2024.

12. Department of Health and Social Care. Roll out of whole care home testing (letter) 2020 [accessed 9 November 2022.

13. UK Cabinet Office. Our plan to rebuild: The UK Government’s COVID-19 recovery strategy 2020 [Available from: <https://www.gov.uk/government/publications/our-plan-to-rebuild-the-uk-governments-covid-19-recovery-strategy> accessed 29 March 2024

14. Department of Health and Social Care. Everyone in the United Kingdom with symptoms now eligible for coronavirus tests 2020 [Available from: <https://www.gov.uk/government/news/everyone-in-the-united-kingdom-with-symptoms-now-eligible-for-coronavirus-tests> accessed 29 March 2024.

15. Department of Health and Social Care. Government to offer antibody tests to health and social care staff and patients in England [Withdrawn] 2020 [Available from: <https://www.gov.uk/government/news/government-to-offer-antibody-tests-to-health-and-social-care-staff-and-patients-in-england> accessed 29 March 2024.

16. Department of Health and Social Care. Roll out of whole care home testing – extension of eligibility (letter) 2020 [Available from: <https://www.nationalcareforum.org.uk/wp-content/uploads/2020/06/2020-06-05_Extension-of-eligibility-for-whole-care-home-testing.pdf> accessed 29 March 2024.

17. Department of Health and Social Care. COVID-19 tests offered to every care home for elderly or those with dementia 2020 [Available from: <https://www.gov.uk/government/news/covid-19-tests-offered-to-every-care-home-for-elderly-or-those-with-dementia> accessed 29 March 2024.

18. Department of Health and Social Care. Whole home testing rolled out to all care homes in England 2020 [Available from: <https://www.gov.uk/government/news/whole-home-testing-rolled-out-to-all-care-homes-in-england> accessed 29 March 2024.

19. Department of Health and Social Care. Regular retesting rolled out for care home staff and residents 2020 [Available from: <https://www.gov.uk/government/news/regular-retesting-rolled-out-for-care-home-staff-and-residents> accessed 29 March 2024.

20. Department of Health and Social Care. Care homes: outbreak testing and regular testing (letter) 2020 [Available from: <https://www.careengland.org.uk/sites/careengland/files/Adult%20Social%20Care%20testing%20strategy%20letter%20to%20DPHs%20and%20DASSs%203.7.20.pdf> accessed 29 March 2024.

21. Department of Health and Social Care. Further information on coronavirus testing in care homes (letter) 2020 [Available from: <https://www.careengland.org.uk/sites/careengland/files/DHSC%20Care%20Home%20Testing%20-%20Letter%2031%20July%202020.pdf> accessed 19 October 2022

22. Department of Health and Social Care. £149 million to support increased care home testing 2020 [Available from: <https://www.gov.uk/government/news/149-million-to-support-increased-care-home-testing> accessed 29 March 2024.

23. Department of Health and Social Care. Changes to coronavirus outbreak testing in care homes (letter) 2021 [Available from: <https://documents.hants.gov.uk/adultservices/Letter-DHSC-Changes-coronavirus-outbreak-testing-care-homes-18-2-21.pdf> accessed 29 March 2024.

24. Department of Health and Social Care. Frontline health and care staff can work rather than self-isolate 2021 [Available from: <https://www.gov.uk/government/news/frontline-health-and-care-staff-can-work-rather-than-self-isolate> accessed 29 March 2024.

25. UK Health Security Agency. COVID-19 care home testing guidance for regular and outbreak testing of staff and residents 2022 [Available from: <https://www.gov.uk/government/publications/coronavirus-covid-19-testing-in-adult-care-homes/covid-19-care-home-testing-guidance-for-regular-and-outbreak-testing-of-staff-and-residents> accessed 29 March 2024.

26. Hica Group. Coronavirus Update – 16th December 2022 2022 [Available from: <https://www.hica-uk.com/covid-19-coronavirus/> accessed 29 March 2024.

27. UK Health Security Agency. Confirmatory PCR tests to be temporarily suspended for positive lateral flow test results 2022 [Available from: <https://www.gov.uk/government/news/confirmatory-pcr-tests-to-be-temporarily-suspended-for-positive-lateral-flow-test-results> accessed 29 March 2024.

28. Croner-i. Guidance updated on Covid-19 testing in adult care homes 2022 [Available from: <https://app.croneri.co.uk/whats-new/guidance-updated-covid-19-testing-adult-care-homes> accessed 20 January 2023.

29. Riley S, Atchison C, Ashby D, et al. REal-time Assessment of Community Transmission (REACT) of SARS-CoV-2 virus: Study protocol. *Wellcome Open Res* 2020;5:200. doi: 10.12688/wellcomeopenres.16228.2 [published Online First: 2021/05/19]

30. Bajaj S, Chen S, Creswell R, et al. Understanding COVID-19 testing behaviour in England through a sociodemographic lens. *medRxiv* 2023:2023.10.26.23297608. doi: 10.1101/2023.10.26.23297608

31. Sanger Institute. COVID–19 Genomic Surveillance nd [Available from: <https://covid19.sanger.ac.uk/lineages/raw> accessed 26 March 2024.

32. UK Health Security Agency (confidential internal document). Care home staff LFD testing research insights playback February 2021, 2021.

33. Zhang X, Barr B, Green M, et al. Impact of community asymptomatic rapid antigen testing on covid-19 related hospital admissions: synthetic control study. *Bmj* 2022;379:e071374. doi: 10.1136/bmj-2022-071374 [published Online First: 2022/11/24]

34. Sandmann FG, Davies NG, Vassall A, et al. The potential health and economic value of SARS-CoV-2 vaccination alongside physical distancing in the UK: a transmission model-based future scenario analysis and economic evaluation. *Lancet Infect Dis* 2021;21(7):962-74. doi: 10.1016/s1473-3099(21)00079-7 [published Online First: 2021/03/22]

35. Department of Health and Social Care, Office for National Statistics, Government Actuary’s Department, et al. Direct and Indirect Impacts of COVID-19 on Excess Deaths and Morbidity: Executive Summary 2020 [Available from: <https://assets.publishing.service.gov.uk/government/uploads/system/uploads/attachment_data/file/918738/S0650_Direct_and_Indirect_Impacts_of_COVID-19_on_Excess_Deaths_and_Morbidity.pdf> accessed 29 March 2024.

36. EY–Oxford Health Analytics Consortium. Evaluation of the national COVID-19 testing programme in England between October 2020 and March 2022 2023 [Available from: <https://assets.ey.com/content/dam/ey-sites/ey-com/en_uk/resources/evaluation-of-covid-19-testing-in-england-eoha-conference-edition.pdf> accessed 26 March 2024.

37. NHS England. National Cost Collection for the NHS nd [Available from: <https://www.england.nhs.uk/costing-in-the-nhs/national-cost-collection/> accessed 29 March 2024.
